# Elevated antioxidant defence in the brain of deep-diving pinnipeds

**DOI:** 10.3389/fphys.2022.1064476

**Published:** 2022-12-16

**Authors:** Gerrit A. Martens, Lars P. Folkow, Thorsten Burmester, Cornelia Geßner

**Affiliations:** ^1^ Institute of Cell and Systems Biology of Animals, University of Hamburg, Hamburg, Germany; ^2^ Department of Arctic and Marine Biology, UiT The Arctic University of Norway, Tromsø, Norway

**Keywords:** marine mammals, hypoxia, antioxidants, oxidative stress, brain, pinniped, seals, positive selection

## Abstract

While foraging, marine mammals undertake repetitive diving bouts. When the animal surfaces, reperfusion makes oxygen readily available for the electron transport chain, which leads to increased production of reactive oxygen species and risk of oxidative damage. In blood and several tissues, such as heart, lung, muscle and kidney, marine mammals generally exhibit an elevated antioxidant defence. However, the brain, whose functional integrity is critical to survival, has received little attention. We previously observed an enhanced expression of several antioxidant genes in cortical neurons of hooded seals (*Cystophora cristata*). Here, we studied antioxidant gene expression and enzymatic activity in the visual cortex, cerebellum and hippocampus of harp seals (*Pagophilus groenlandicus*) and hooded seals. Moreover, we tested several genes for positive selection. We found that antioxidants in the first line of defence, such as superoxide dismutase (SOD), glutathione peroxidase (GPX) and glutathione (GSH) were constitutively enhanced in the seal brain compared to mice (*Mus musculus*), whereas the glutaredoxin and thioredoxin systems were not. Possibly, the activity of the latter systems is stress-induced rather than constitutively elevated. Further, some, but not all members, of the glutathione-s-transferase (GST) family appear more highly expressed. We found no signatures of positive selection, indicating that sequence and function of the studied antioxidants are conserved in pinnipeds.

## Introduction

Marine mammals have undergone a fascinating transition from a terrestrial to a marine habitat and evolved various adaptations to aquatic life. One of the major challenges is the supply of oxygen while foraging at depth. During diving, breathing, and consequently the intake of oxygen, stops. Marine mammals have adapted by evolving a high capacity for oxygen storage, e.g., high levels of muscle myoglobin and an elevated blood volume with a high content of hemoglobin (e.g., Ponganis, 2011; Blix, 2018). Additionally, bradycardia and peripheral vasoconstriction during dives contribute to an efficient use of stored oxygen (Scholander, 1940; Ponganis, 2011; Blix 2018). In spite of these preventive adaptations, deep-diving seals can experience very low blood oxygen tensions. During long, voluntary dives the deep-diving Weddell (*Leptonychotes weddellii*) and northern elephant (*Mirounga angustirostris*) seals endure arterial oxygen tensions well below 20mmHg (Qvist et al., 1986; Meir et al., 2009).

In addition to the limited availability of oxygen when submerged, transformation of oxygen into reactive oxygen species (ROS) also represents a major challenge. ROS are radical or non-radical oxygen species produced by the partial reduction of oxygen. Mitochondria have been recognized as an important intracellular source of ROS, which can arise during oxidative phosphorylation that produces energy in the form of ATP. ATP production is accomplished by a tetravalent reduction of oxygen. In normal physiological conditions, 1–4% of the oxygen is incompletely reduced and leaks from the electron transport chain (ETC) in the form of superoxide radical (O_2_^•–^) (Kevin et al., 2005). However, ROS are also produced in the endoplasmatic reticulum, peroxisomes, lysosomes and others (Milkovic et al., 2019). ROS play an important role as redox signaling messengers contributing, amongst others, to cell proliferation and survival and thus, are part of the normal functioning of cells. However, when ROS are produced in excess, signaling ability is lost and macromolecules are unspecifically damaged promoting several pathologies such as neurodegenerative diseases, atherosclerosis, diabetes and cancer (Ray et al., 2012; Milkovic et al., 2019). Acknowledging both the importance and potential risks of ROS, physiological levels of ROS can be termed oxidative eustress, while excessive oxidant challenge may be considered as oxidant distress (Sies et al., 2017; Sies, 2021).

Diving bouts of marine mammals lead to recurrent phases in which the availability of oxygen is limited, followed by reoxygenation upon resurfacing. When the animal surfaces, all tissues are reperfused with oxygenated blood (e.g., Blix, 2018). It is known that cellular hypoxia leads to a reduced activity of complex IV (cytochrome oxidase) in the ETC and that re-introduction of oxygen causes an accelerated leakage of radicals from more proximal complexes and the production of O_2_^•–^ is increased (Kevin et al., 2005). In terrestrial organisms, ischemia/reperfusion increases ROS production and the potential for oxidative damage (Halliwell and Gutteridge, 2015). In marine mammals, reoxygenation upon resurfacing replenishes oxygen stores and boosts aerobic ATP production, but it may also generate ROS and oxidative stress can occur (Fridovich, 1998). Evidence exist to indicate that marine mammals and terrestrial, hibernating species, such as some bats and ground squirrels, display adaptations to fast reoxygenation that prevent reperfusion injury (Hermes-Lima et al., 2015). In ringed seals, ischemia and subsequent reoxygenation occurring during and after a dive increased ROS production, but not the oxidative stress (Zenteno-Savín and Elsner, 1998; Zenteno-Savín and Elsner, 2000).

To prevent oxidative stress, organisms have evolved an antioxidant defence consisting of enzymes and non-enzymatic antioxidants, such as glutathione (GSH), uric acid, melatonin, vitamins C and E and others (Milkovic et al., 2019). Prominent examples of antioxidant enzymes that were analysed in this study are the superoxide dismutase (SOD) converting O_2_^•–^ into hydrogen peroxide (H_2_O_2_), and glutathione peroxidase (GPX) transforming H_2_O_2_ into H_2_O and limiting the hydroxyl radical (OH^•^) formation. Glutathione-S-transferase (GST) binds toxic products to glutathione and the resulting glutathione conjugates can then be removed from the organism (Cooper and Kristal, 1997). Moreover, we studied representatives of the glutaredoxin and thioredoxin systems that play a key role in antioxidant defence and redox state of a cell. Amongst others, these systems remove ROS or activate oxidative-sensitive transcription factors (Lu and Holmgren, 2014).

Considering the diving behavior of marine mammals, one might expect these species to have a constitutively higher antioxidant defence system in adaptation to an elevated risk of ROS exposure. Indeed, previous studies in some pinnipeds, the manatee (*Trichechus manatus*) and several cetacean species, have generally revealed higher antioxidant levels in diving compared to non-diving mammals (Elsner et al., 1998; Filho et al., 2002; Zenteno-Savın et al., 2002; Vázquez-Medina et al., 2006; Vázquez-Medina et al., 2012). In these studies, blood samples, but also samples from heart, lung, kidney, liver and skeletal muscle, were studied. To the best of our knowledge, with exception of a study in the bottlenose dolphin (*Tursiops truncatus*) and the dwarf sperm whale (*Kogia sima*) (Cantú-Medellín et al., 2011), the antioxidant status of the diving brain has not previously been studied. Since the functional integrity of the brain is essential to the survival of an organism, we expect marine mammals to have an elevated cerebral antioxidant defence to prevent oxidative stress. In a comparative transcriptomic analysis of neurons of the visual cortex in hooded seals (*Cystophora cristata*) and mice (*Mus musculus*), we previously observed a significantly higher expression of antioxidant genes in hooded seals (Geßner et al., 2022). Similarly, in the visual cortex of whales, we found a high expression of transcripts related to the detoxification of ROS when compared to cattle (*Bos taurus*) (Krüger et al., 2020).

In this study, we extend the results of Geßner et al. (2022) and aim to identify whether elevated antioxidant levels found in neurons of the visual cortex are unique to this brain region or if they are present in other regions and could, thus, possibly be representative for the whole brain. Further, we studied antioxidant levels in harp seals (*Pagophilus groenlandicus*), to investigate whether an increased antioxidant defence of the brain might be relevant in pinniped species other than the hooded seal. We studied antioxidant gene expression in the visual cortex, cerebellum and hippocampus of hooded seals, harp seals and mice. Further, we determined the enzymatic activity of SOD, GST, GPX and glutathione reductase (GSR) and the concentration of reduced glutathione (GSH) in these brain regions. We further tested a set of antioxidant genes in seven pinniped species for positive selection, to test whether selection pressure has favoured changes in gene sequences that might also lead to functional changes.

## Methods

### Antioxidant gene expression in neurons of the visual cortex

Transcriptomes of mouse and hooded seal neurons that were separated *via* laser-capture microdissection of the visual cortex were available from Geßner et al. (2022). The expression analysis *via* RNA-seq and the differential expression analysis were performed as described in Geßner et al. (2022), using the CLC workbench v.10.0.1. Briefly, quality-trimmed reads (Phred score >35, removal of first 20 5′-terminal nucleotides, with less than two ambiguous bases and reads >30 nucleotides in length) were mapped against the human genome (assembly GRCh38. p13) that served as a reference genome. Only reads that matched 75% of the read length and 75% of the nucleotides to the reference genome were included in the mapping. Gene expression is presented as TPM (Transcripts Per Kilobase Million mapped reads), whereby only reads that mapped uniquely in the genome were included in the calculation of TPM values. *p*-values of differentially expressed genes were corrected for multiple testing using the false discovery rate (FDR) (Benjamini and Hochberg, 1995). Only genes with a p_FDR_ ≤ 0.05, TPM-value ≥1 in either species and a fold change (FC) ≥ 2 or ≤−2 were considered as significantly differentially expressed genes (DEGs). From these DEGs, genes of the GO terms “antioxidant activity” (GO:0016209) were taken from Geßner et al. (2022). For this study, we additionally extracted genes of “glutathione metabolic process” (GO:0006749) (http://www.informatics.jax.org) and a list of human antioxidant genes (Gelain et al., 2009). Additionally, we included heme oxygenase 2 (*HMOX2*) and Paraoxonase 2 (*PON2*), which are both known to be involved in the antioxidant defence (Barañano et al., 2002; Ng et al., 2006).

### Animals

Hooded seals (*Cystophora cristata*; *n* = 4 adult females, March 2019) and harp seals (*Pagophilus groenlandicus*; *n* = 3 adult females in March 2018; *n* = 1 adult female in March 2019, no hippocampus available for latter individual) were captured in the pack ice of the Greenland Sea under permits from relevant Norwegian and Greenland authorities. The hooded seals were euthanized immediately following live-capture, by sedation with an intramuscular injection of zolazepam/tiletamine (1.5–2.0 mg per kg of body mass), followed by catheterization of the extradural intravertebral vein and i. v. injection of an overdose of pentobarbital (Euthasol vet. Le Vet B.V. Netherlands; ∼30 mg per kg of body mass). The harp seals were all shot to the head and bled, after which brain tissue was immediately sampled from intact brain regions. For the repetition of the GST and GSH/GSSG assays that were performed at a later point in time, hooded seal tissues (from *n* = 3 adult females) were collected in March 2021, using the same procedure as described above for this species. All animal handling was in accordance with the Norwegian Animal Welfare Act and with approvals from the Norwegian Food Safety Authority (permits no. 12268 and 22451). Adult female mice (C57BL/6, *n* = 20, whereby *n* = 4 were used per assay and qPCR) were a gift by Prof. Dr. Christian Lohr (University of Hamburg, Hamburg, Germany) and were anaesthetized with 1 ml isoflurane (Forene, Abbott, Germany) in a chamber (1,000 ml) and decapitated. All animals were handled according to the EU Directive 63 (Directive 2010/63/EU). This mouse strain has served as model organism in studies investigating oxidative stress, e.g., during aging (Jeong et al., 2018) or when exposed to ethanol during brain development (Kumral et al., 2005). C57BL/6 mice were also used in a previous comparative study with diving mammals (Geßner et al., 2022). While mouse strains and their hybrids differ in susceptibility to hypoxia, C57BL/6 appear to be an intermediate type, neither particularly sensitive nor tolerant to hypoxia (Sheldon et al., 1998). Fresh tissue of the visual cortex, cerebellum and hippocampus was frozen in liquid nitrogen and later transferred to -80°C for storage until subsequent use.

### Quantitative real-time reverse transcription polymerase chain reaction (qPCR)

For expression analyses using qPCR, we selected essential antioxidant genes (*GPX3, SOD1, GSTK1, GSTO1*) or genes that represent a component of important antioxidant systems (*TXNRD3, GLRX2*). Only genes with a p_FDR_ ≤ 0.05, TPM-value ≥1 in either species and a fold change (FC) ≥ 3 or ≤−3 in the transcriptomic data were considered for qPCR. Since we aim to determine the antioxidant defence of neurons, we used *RBFOX3* as a neuronal marker to account for different numbers of neurons in every tissue sample. Primer sequences (Supplementary Table S1A) for the mouse were designed based on sequences retrieved from GenBank (https://www.ncbi.nlm.nih.gov/genbank/) and hooded seal sequences were extracted from Geßner et al. (2022). Since there are no harp seal data available on GenBank, primer from the hooded seal were used and primer specificity verified *via* gel electrophoresis and/or sequencing.

Total RNA from frozen tissue samples of the visual cortex, cerebellum and hippocampus of hooded seals, harp seals and mice were extracted using the Crystal RNA Mini Kit (BiolabProducts, Gödenstorf, Germany) according to the manufacturer’s instructions, including an on-column DNA digestion with RNase-free DNase (Qiagen, Germany). The quantity and integrity of the isolated total RNA were assessed using the Agilent 4,200 TapeStation System and RNA ScreenTape Assay (Agilent Technology, Santa Clara, United States). First-strand cDNA was synthesized from 1 µg of total RNA with Oligo (dT)_18_ primer using the RevertAid H Minus First Strand cDNA Synthesis Kit (Thermo Scientific, Germany). The qPCR was performed with a 7,500 Fast Real-Time PCR System and the Power SYBR Green master mix (Applied Biosystems, Darmstadt, Germany) using a standard PCR protocol (step 1–2: 50°C for 2 min, 95°C for 10 min, step 3–5: 95°C for 30 s, 58°C for 30 s, and 72°C for 30 s; 40 cycles step 3–5). Primer efficiencies (Supplementary Table S1B) were assessed with serial dilutions of pooled cDNA samples from each brain region and species, respectively. For relative comparisons of gene expression, a 1:25 dilution of the cDNA (equivalent to 40 ng RNA) was used per reaction. The experiments, including negative controls, were carried out as triplicates. To account for variations between runs, identical interrun calibrators were added on each microtiter qPCR plate, with pooled cDNA from visual cortex of each species, respectively. Dissociation curve analyses were used to validate the specificity of the amplifications. Raw Ct-values were calculated with the 7,500 System Sequence Detection Software 2.0.6 (Applied Biosystems) and adjusted according to interrun calibrators. The dCt values were obtained by normalizing the Ct-values to the widely used neuronal marker *RBFOX3* encoding NeuN protein (Duan et al., 2016). Fold changes (FC) for harp and hooded seals were calculated with mouse samples as reference using the ddCt method. Statistical analysis was performed on dCt values using the statistical program R version 4.1.2 (R Core Team, 2013) and the Tukey_hsd function of the rstatix_0.7.0 package (Kassambara, 2021). Fold changes were visualized with the ggpubr package (Kassambara, 2020).

### Enzymatic activity assays

For all assays, ∼20 mg of tissue from every brain region was washed twice in phosphate buffered saline (PBS) (140 mM NaCl, 2.7 mM KCl, 8.1 mM Na_2_HPO_4_, 1.5 mM KH_2_PO_4_, pH7.4) to remove blood before assay-specific buffers were used for homogenization.

#### Glutathione peroxidase (GPX) activity

Total GPX activity was determined using the Glutathione Peroxidase Assay Kit (Cayman Chemical, item no. 703102). Tissue samples (20 mg) were homogenized in 75 µL cold buffer (50 mM Tris-HCl, pH7.5, 5 mM EDTA and 1 mM DTT) according to the manufacturer’s instructions. Total protein contents were measured with the Bradford assay (Carl Roth, Karlsruhe, Germany) and adjusted with homogenization buffer to the sample with the lowest concentration (17.94 mg/ml). Of all adjusted samples a 1:5 dilution was prepared and used in the assay. Absorbance was read every minute at 340 nm using a DTX 880 Multimode Detector (Beckmann Coulter, Krefeld, Germany). The decrease in absorbance was measured for 25 min. The decrease was linear up until minute 10 and thus, the first 10 data points were used for statistical analysis and to calculate the GPX-activities (nmol/min/ml) according to the manufacturer’s instructions.

#### Superoxide dismutase (SOD) and glutathione-S-transferase (GST) activity

The tissues samples (20 mg) were homogenized in 100 µL of cold 20 mM HEPES buffer (1 mM EDTA, 210 mM mannitol and 70 mM sucrose, pH 7.2). Total protein concentration was measured with the Bradford assay (Carl Roth, Karlsruhe, Germany) and adjusted with HEPES buffer to the sample with the lowest concentration (1.97 mg/ml).

##### SOD activity

All samples were further diluted 1:300 in HEPES buffer. The total SOD activity was measured with the Superoxide Dismutase Assay Kit (Cayman Chemical, item no. 706002) according to the manufacturer’s instructions. The absorbance was determined at 450 nm and SOD activities are expressed as U/ml.

##### GST activity

All samples were diluted 1:5 in HEPES buffer. The total GST activity was determined using the Glutathione S-Transferase Assay Kit (Cayman Chemical, item no. 703302) according to the manufacturer’s instructions. The absorbance was recorded every minute at 340 nm for 60 min and the linear range from minute 1 to 9 was used for calculation of the GST activities (nmol/min/ml). Measurement of the GST activity was repeated using a different GST assay kit (Abcam, ab65325) and newly sampled hooded seal tissues (March 2021). The tissue was homogenized in Assay buffer and the concentration of all samples was adjusted to 3.9 mg/ml. The assay was performed according to the manufacturer’s instruction using a 1:5 dilution of the samples.

#### Glutathione reductase (GSR) activity

The tissues samples (20 mg) were homogenized in 100 µL of cold buffer (50 mM potassium phosphate, pH 7.5, 1 mM EDTA). Total protein concentration was measured with the Bradford assay (Carl Roth, Karlsruhe, Germany) and adjusted with Sample buffer (Cayman Chemical, item no. 703202) to the sample with the lowest concentration (2.3 mg/ml). The GSR activity was measured according to the manufacturer’s instructions using the Glutathione Reductase Assay kit (Cayman Chemical, item no.703202). The absorbance was read at 340 nm once every minute for 6 min and GSR activity was calculated in nmol/min/ml.

#### GSH/GSSG ratio

The ratio of reduced glutathione (GSH) and oxidized glutathione (GSSG) was determined using the Amplite Fluorometric Glutathione GSH/GSSG Ratio Assay Kit Green Fluorescence (Biomol, Catalog number 10056). Tissues were homogenized in HEPES buffer, protein concentrations adjusted to 5.2 mg/ml and diluted 1:50 in Assay buffer. The assay was run according to manufacturer’s instructions. The results were verified at a later point in time using a different assay, the GSH/GSSG Ratio Detection Assay Kit (Abcam, ab138881), and newly sampled hooded seal tissues from March 2021. Tissues were homogenized in 1xPBS (pH = 6) with 0.5% NP40 (Abcam, ab142227). Protein concentrations were adjusted to 3 mg/ml and samples were diluted 1:50 in Assay buffer. Before both assays were run, samples were deproteinized with the ReadiUse™ TCA Deproteinization Sample Preparation Kit (Biomol, ABD-19501).

### Statistical analysis of enzymatic activity assays

The statistical analyses were carried out in the R v. 3.5.1 statistics program (R Core Team, 2013). Significant differences in the means of our species and brain regions were identified with an ANOVA, since the residuals of all assays were normally distributed. We employed the Levene test included in the Rcmdr package (Fox et al., 2019) to test for variance homogeneity. Then, the Tukey-Kramer test of the DTK package (Lau, 2013) was used to test for significant differences between species and brain regions. To correct for type I errors, *p*-values were corrected with the False Discovery Rate (Benjamini and Hochberg, 1995) using the p. adjust ()-function in R. Results are presented as mean values ±SEM.

### Inferring positive selection

We tested for positive selection in all genes explored *via* qPCR, that is GPX3, SOD1, GSTK1 GSTO1, TXNRD3, GLRX2 and additionally GSR. We explored selection pressures in seven pinnipeds and in five terrestrial carnivores that served as non-diving relatives. Among pinnipeds, the deep diving hooded seal (*Cystophora cristata*), the Weddell seal (*Leptonychotes weddelli*), the southern elephant seal (*Mirounga leonina*), the Hawaiian monk seal (*Neomonachus schauinslandi*), the gray seal (*Halichoerus grypus*), the harbour seal (*Phoca vitulina*) and the walrus (*Odobenus rosmarus divergens*) were compared with the dog (*Canis lupus familiaris*), ferret (*Mustela putorius furo*), giant panda (*Ailuropoda melanoleuca*), grizzly bear (*Ursus arctos horribilis*) and the polar bear (*Ursus maritimus*). The nucleotide sequences were retrieved from GenBank (Supplementary Table S3 for accession numbers) except for the sequences of the hooded seal, that were extracted from Geßner et al. (2022). For each gene, nucleotide sequences were aligned using TranslatorX (http://translatorx.co.uk, 21.01.2022) providing a peptide alignment generated in MAFFT (Katoh and Standley, 2013) to ensure alignment quality. Selection pressure was assessed by estimating the non-synonymous to synonymous rate ratio using the Branch-wide Unrestricted Statistical Test for Episodic Diversification (BUSTED) (Murrell et al., 2015) and the adaptive Branch-Site Random Effects Likelihood (aBSREL) model (Smith et al., 2015) on the Datamonkey server (https://www.datamonkey.org, 21.01.2022) (Pond and Frost, 2005). In both models, pinnipeds were denoted as foreground branches in which some sites might be positively selected, whereas non-diving mammals served as background branches in which positive selection is absent. BUSTED assesses whether a gene has experienced positive selection in at least one site in at least one of the branches tested, while aBSREL estimates for every foreground branch whether a proportion of sites has undergone positive selection. Only genes for which positive selection was inferred by both methods were considered positively selected.

## Results

### Antioxidant gene expression in neurons of hooded seals and mice

We extracted the antioxidant gene expression values from the cell-type specific transcriptome of visual cortex neurons in hooded seals and mice (Geßner et al., 2022). We found a total of 49 differentially expressed antioxidant genes (DEGs), i.e. genes with p_FDR_ ≤ 0.05, a TPM-value ≥1 in either species and a fold change (FC) ≥ 2 or ≤−2. Table 1 lists genes that were further analysed by e.g. qPCR in this study. Please see Supplementary Table S2 for a complete list of all 49 genes.

**TABLE 1:** Antioxidant gene expression in visual cortex neurons of hooded seals (*Cystophora cristata*), expressed in relation to gene expression in mice (*Mus musculus*), with a p_FDR_ ≤ 0.05, TPM-value ≥1 and a fold change ≥2 or ≤ -2. p_FDR_ represents the *p*-value corrected for multiple testing using the False Discovery Rate (FDR), while Transcripts Per Kilobase Million mapped reads (TPM) represent normalized expression values. Only genes analysed in qPCR, and/or for which enzyme activity assays were available, are presented. For a complete list, please see Supplementary Table S2.

Annotated term	Gene symbol	Fold change	FDR	TPM mouse	TPM hooded seal
antioxidant activity (GO:0016209) adapted from Geßner et al. (2022)					
glutathione peroxidase activity	*GPX1*	2.2	2.52∙10^−5^	65.4	140.2
	*GPX3*	8.6	1.47∙10^−11^	4.0	34.2
	*GSTK1*	26.9	6.76∙10^−72^	0.7	19.1
	*GSTO1*	15.1	2.30∙10^−30^	3.6	53.9
	*GSTO2*	9.9	2.12∙10^−7^	0.1	1.1
antioxidant activity	*SOD1*	9.1	7.29∙10^−59^	52.8	481.0
glutathione-disulfide reductase (NADPH) activity	*GSR*	2.0	1.50∙10^−2^	6.5	12.8
thioredoxin-disulfide reductase activity	*TXNRD3*	4.7	6.03∙10^−7^	1.3	5.9
Human antioxidant genes (Gelain et al., 2009)					
Thiol redox	*GLRX2*	−3.3	1.09∙10^−8^	3.8	1.1

Of the 49 genes, 28 were more highly expressed in hooded seal compared to mouse neurons. The annotated GO term “glutathione peroxidase activity” was represented by eight of these 28 genes and thus, was the most frequently represented term. For example, genes assigned to this function were the glutathione peroxidase 1 (*GPX1*, FC = 2.2) and 3 (*GPX3*, FC = 8.6) and the glutathione-S-transferase kappa 1 (*GSTK1*, FC = 26.9), omega 1 (*GSTO1*, FC = 15.1), omega 2 (*GSTO2*, FC = 9.9) and the microsomal glutathione-S-transferase 1 (*MGST1*, FC = 13.7).

The GO term “antioxidant activity” was represented by six genes and all of them were more highly expressed in neurons of hooded seals compared to mice. Among these genes were S100 calcium binding protein A9 (*S100A9*, FC = 99.7, but we note that TPM values were relatively low in both species), superoxide dismutase 1 (*SOD1*, FC = 9.1), peroxiredoxin-like 2 A (*PRXL2A*, FC = 3.5), peroxiredoxin 2 (*PRDX2*, FC = 3.4) and selenoprotein W (*SELENOW*, FC = 2). Further, within antioxidant genes, *SOD1* was among the top five genes with the highest TPM value (TPM = 481) in hooded seal neurons, followed by *PRDX2* (TPM = 388), *GPX1* (TPM = 140), heme oxygenase 2 (*HMOX2*, TPM = 99) and glyoxalase 1 (*GL O 1*, TPM = 70). Even when all genes (not only antioxidants) in the transcriptome were considered, SOD1 had a high expression, being the gene with the 22nd highest TPM value. Antioxidant genes with the top five F C were S100A9 (FC = 99.7), *GSTK1* (FC = 26.9), arachidonate 5-lipoxygenase activating protein (ALOX5AP, FC = 21.5), selenoprotein T (SELENOT, FC = 17.6) and GSTO1 (FC = 15.1).

### Antioxidant gene expression in brain regions of seals and mice *via* qPCR

**FIGURE 1:**
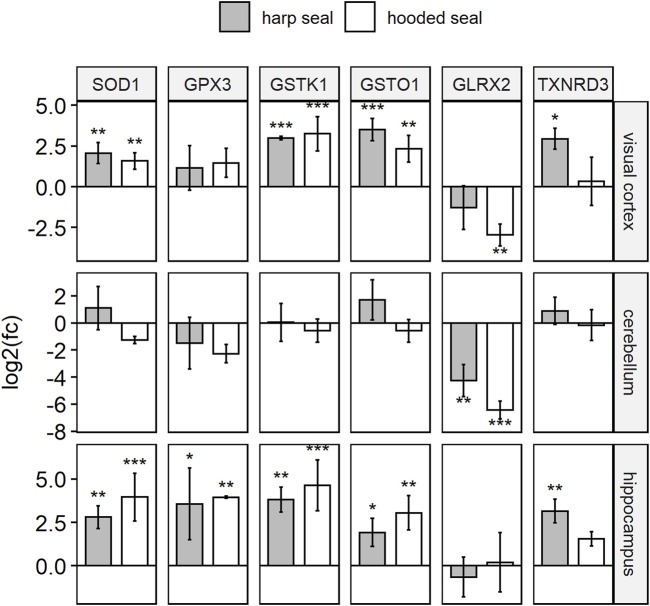
Antioxidant gene expression of hooded seals and harp seals, when compared to mice, as determined using qPCR. Relative gene expression is presented in log2 fold changes [log2 (fc)], whereby positive/negative values represent higher/lower expression in seals as compared to mice. Significance is expressed by asterisks (*p* ≤ 0.05 (*), *p* ≤ 0.01 (**), *p* ≤ 0.01 (***).

In order to test whether elevated antioxidant gene expression is also present in other brain regions and in diving mammals other than the hooded seal, we performed qPCR analyses in the visual cortex, the cerebellum and the hippocampus of hooded seals and harp seals, and compared results to mice (Figure 1).

In the visual cortex, expression of all six antioxidants confirmed the transcriptomic data, i.e. the expression was higher in hooded seals than in mice (p_GSTK1_ = 0.0003, p_GSTO1_ = 0.0033, p_SOD1_ = 0.0038), although the difference in expression was not always significant (GPX3 and TXNRD3). As in the transcriptomic data, GLRX2, was less expressed in hooded seals than in mice (p_GLRX2_ = 0.002). Similarly, expression in harp seals was significantly (p_GSTO1_ = 0.0004, p_SOD1_ = 0.0011, p_TXNRD3_ = 0.0112) or insignificantly higher (GPX3, GSTK1) and insignificantly lower for GLRX2.

In the cerebellum, GLRX2 was less expressed in hooded seals (*p* = 0.0008) and harp seals (*p* = 0.0087) than in mice. There were no significant differences in the expression of the other antioxidants between species.

In the hippocampus, we observed no difference in GLRX2 expression. However, the other antioxidants were all significantly more highly expressed in hooded seals (p_GPX3_ = 0.0092, p_GSTK1_ = 0.0008, p_GSTO1_ = 0.0019, p_SOD1_ = 0.0002) and harp seals (p_GPX3_ = 0.0155, p_GSTK1_ = 0.0025, p_GSTO1_ = 0.0221, p_SOD1_ = 0.0071, p_TXNRD3_ = 0.0051) with exception of an insignificantly higher expression of TXNRD3 in hooded seals.

### Enzymatic activity assays

#### Elevated SOD activities in pinniped brains compared to mice

**FIGURE 2:**
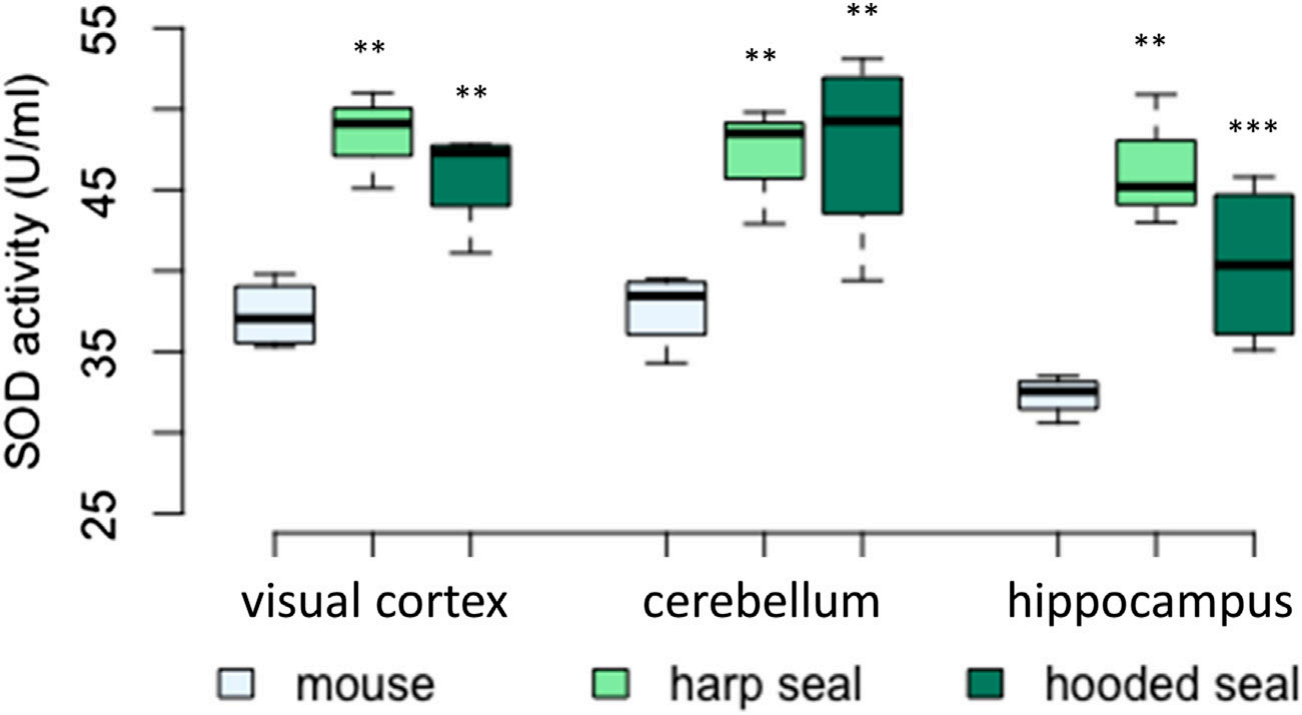
Superoxide dismutase (SOD) activity (U/ml) in mice, harp seals and hooded seals. Significance levels refer to differences compared to mice in the respective brain region and are represented by asterisks (*p* ≤ 0.05 (*), *p* ≤ 0.01 (**), *p* ≤ 0.01 (***).

In all three brain regions tested (visual cortex, cerebellum, hippocampus), SOD activity was significantly higher in hooded seals and harp seals than in mice (Figure 2; Table 2). In the visual cortex, we observed similar mean rates of 48.4 ± 1.7 U/ml (*p* = 0.003) and 45.9 ± 1.6 U/ml (*p* = 0.006) in the harp and hooded seal, respectively, while mice showed a mean activity of 37.3 ± 1.1 U/ml. The cerebellum exhibited SOD activities similar to the visual cortex, with harp and hooded seals reaching 47 ± 2.1 U/ml (*p* = 0.006) and 47.7 ± 3 U/ml (*p* = 0.003) compared to mice, which showed a mean activity of 37.6 ± 1.2 U/ml. The hippocampus displayed the overall lowest SOD activity levels, with 46.4 ± 2.4 U/ml (*p* ≤ 0.001) and 40.4 ± 2.6 U/ml (*p* = 0.007) in harp and hooded seals, respectively, and 32.3 ± 0.6 U/ml in mice.

**TABLE 2:** Summary of results from the neuronal transcriptome of hooded seals and mice, the gene expression of harp seals (Pgr), hooded seals (Ccr) whole brain tissue normalized to a neuronal marker, as measured using qPCR, the enzymatic activity or concentration levels (for GSH), and the results from tests to infer positive selection. Fold changes (FC) and log2(FC) provide a measure of the difference between pinnipeds and mice, whereby positive values indicate a higher expression/activity/concentration in seals compared to mice. Enzymatic activity of SOD was measured in U/ml, GPX, GST, and GSR in nmol/min/ml and GSH in µM and the difference is presented in the respective unit.

	Transcriptome	Gene expression [qPCR, log2(FC)]						Enzymatic activity (difference to mice)						Pos. Selection
	Fold change (FC)	Visual cortex		Cerebellum		Hippocampus		Visual cortex		Cerebellum		Hippocampus		
		*Pgr*	*Ccr*	*Pgr*	*Ccr*	*Pgr*	*Ccr*	*Pgr*	*Ccr*	*Pgr*	*Ccr*	*Pgr*	*Ccr*	
superoxide dismutase														
SOD1	9.1	2.06	1.58	1.12	−1.26	2.81	3.98	11.6	8.6	9.5	11.5	14.1	8.1	no
glutathione peroxidase														
GPX3	8.6	1.16	1.46	−1.49	−2.28	3.58	3.97	147.8	144.6	225.2	118.6	214.2	146.4	no
glutathione-S-transferases														
GSTK1	26.9	1.16	3.26	−1.49	−0.57	3.58	4.66	−67.8	−71.3	−97.4	−92.7	−65.3	−77.3	no
GSTO1	15.1	3.51	2.34	1.71	−0.58	1.93	3.07							no
glutaredoxin system														
GLRX2	−3.3	−1.29	−2.97	−4.27	−6.45	−0.65	0.19							no
thioredoxin system														
TXNRD3	4.7	2.95	0.33	0.89	−0.16	3.17	1.56							no
glutathione cycle														
GSR	2							9.25	−10	−65.1	86.1	−0.53	−16.6	no
GSH								62.3	69.2	74.1	64.2	56	69.8	

#### Higher GPX activities in pinniped brains compared to mice

**FIGURE 3:**
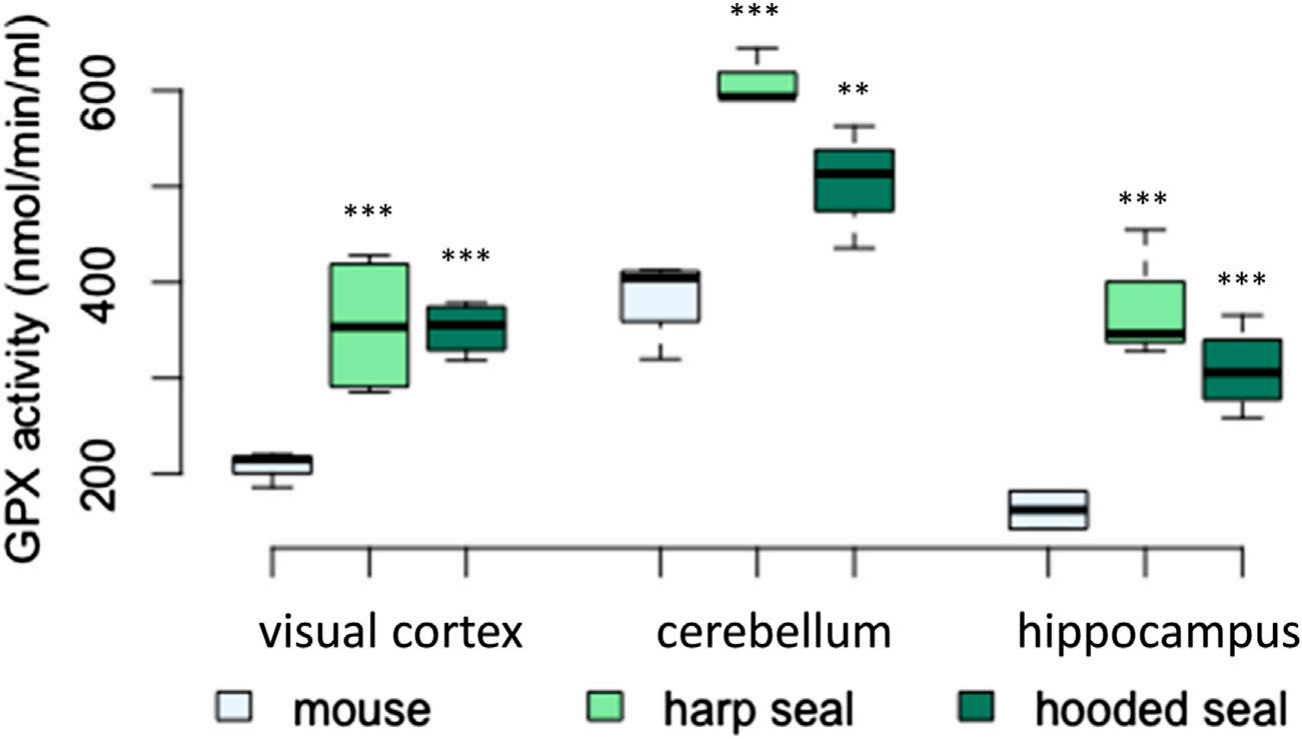
Glutathione peroxidase (GPX) activity (nmol/min/ml) in mice, harp seals and hooded seals. Significance levels refer to differences compared to mice in the respective brain region and are represented by asterisks (*p* ≤ 0.05 (*), *p* ≤ 0.01 (**), *p* ≤ 0.01 (***).

The total GPX activity was significantly higher in hooded seals and harp seals than in mice across brain regions (Figure 3; Table 2). Thus, while the GPX activity in the visual cortex of mice was 207 ± 10.7 nmol/min/ml, hooded and harp seals had similarly higher activities of 351 ± 13.9 nmol/min/ml (*p* = < 0.001) and 355 ± 37.2 nmol/min/ml (*p* = < 0.001), respectively. Across species, the cerebellum displayed the highest GPX activity, with mice reaching 385 ± 22.1 nmol/min/ml, while hooded seals displayed a significantly higher activity of 504 ± 37.1 nmol/min/ml (*p* = 0.003) whereas the highest activity of 610 ± 17 nmol/min/ml was noted in the harp seal cerebellum (*p* < 0.001). In contrast, the hippocampus showed the overall lowest GPX activity, where mice had a lower rate (162 ± 11.2 nmol/min/ml) than either the hooded seal or the harp seal (309 ± 22.2 nmol/min/ml (*p* = < 0.001) and 376 ± 39.4 nmol/min/ml (*p* = 0.001), respectively). With exception of the visual cortex, harp seals displayed the overall highest GPX activity levels, even compared to hooded seals, in both the cerebellum (*p* = 0.007) and the hippocampus (*p* = 0.023).

#### GSR and GST enzyme activities were not higher in pinnipeds than in mice

**FIGURE 4:**
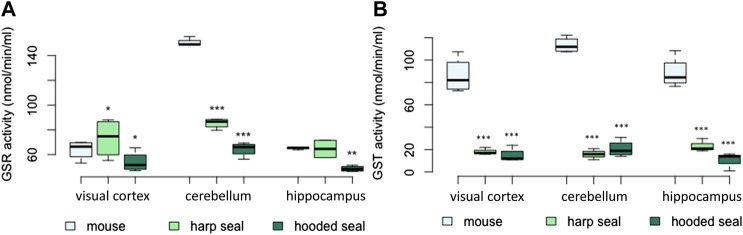
**(A)** Glutathione-disulfide reductase (GSR) activity (nmol/min/ml) and **(B)** glutathione-S-transferase (GST) activity (nmol/min/ml) in mice, harp seals and hooded seals. Significance levels refer to differences compared to mice in the respective brain region and are represented by asterisks (*p* ≤ 0.05 (*), *p* ≤ 0.01 (**), *p* ≤ 0.01 (***).

The results of the GSR enzymatic activity assay were mixed, but overall pinnipeds displayed a lower activity than mice (Figures 4A; Table 2). Enzyme activity was higher in the visual cortex of harp seals (73 ± 8 nmol/min/ml, *p* = 0.03), while hooded seals showed significantly lower activity (54 ± 4.1 nmol/min/ml, *p* = 0.03), compared to mice (64 ± 3.1 nmol/min/ml). In the cerebellum, harp seals (85 ± 2 nmol/min/ml, *p* < 0.001) and hooded seals (64 ± 2.9 nmol/min/ml, *p* < 0.001), had significantly lower enzyme activities than mice (150 ± 1.6 nmol/min/ml). In the hippocampus, we found no difference between harp seals (65 ± 7 nmol/min/ml) and mice (65 ± 0.6 nmol/min/ml), while hooded seals showed significantly lower GSR activities (49 ± 1.1 nmol/min/ml, *p* = 0.01).

The GST activity was measured using two assays. The results were similar and thus, only the results of the Glutathione S-Transferase Assay Kit (Cayman Chemical) are presented. Total GST activity did not confirm the significantly higher expression of GST observed in the neuronal transcriptomic data (Figure 4B). Across brain regions, harp and hooded seals exhibited a significantly lower GST activity compared to mice. While mice had GST activities of 86 ± 7.9, 113 ± 3.5 and 88 ± 6.9 nmol/min/ml in the visual cortex, cerebellum and hippocampus, respectively, we found enzyme activities to be as low as 18 ± 1.8, 16 ± 2.9, and 23 ± 3.4 nmol/min/ml, respectively, in harp seals. Similarly, hooded seals displayed GST activities of 15 ± 3.1, 21 ± 3.7, and 11 ± 3.4 nmol/min/ml in the same brain regions.

#### Higher GSH-levels in pinnipeds compared to mice

The results of two different assays were similar and thus, only the results of the Amplite Fluorometric Glutathione GSH/GSSG Ratio Assay are presented. In order to calculate the GSH/GSSG ratio, both the amounts of reduced glutathione (GSH) and total glutathione (GSH + GSSG) must be determined, whereby the concentration of total glutathione is expected to be equal to or higher than the GSH amount. However, total glutathione levels (GSH + GSSG) of both assays were lower than GSH concentrations, in spite of repeated trials with different sample preparation and different individuals used. Therefore, GSSG determination failed and the specific GSH/GSSG ratio could not be calculated.

**FIGURE 5:**
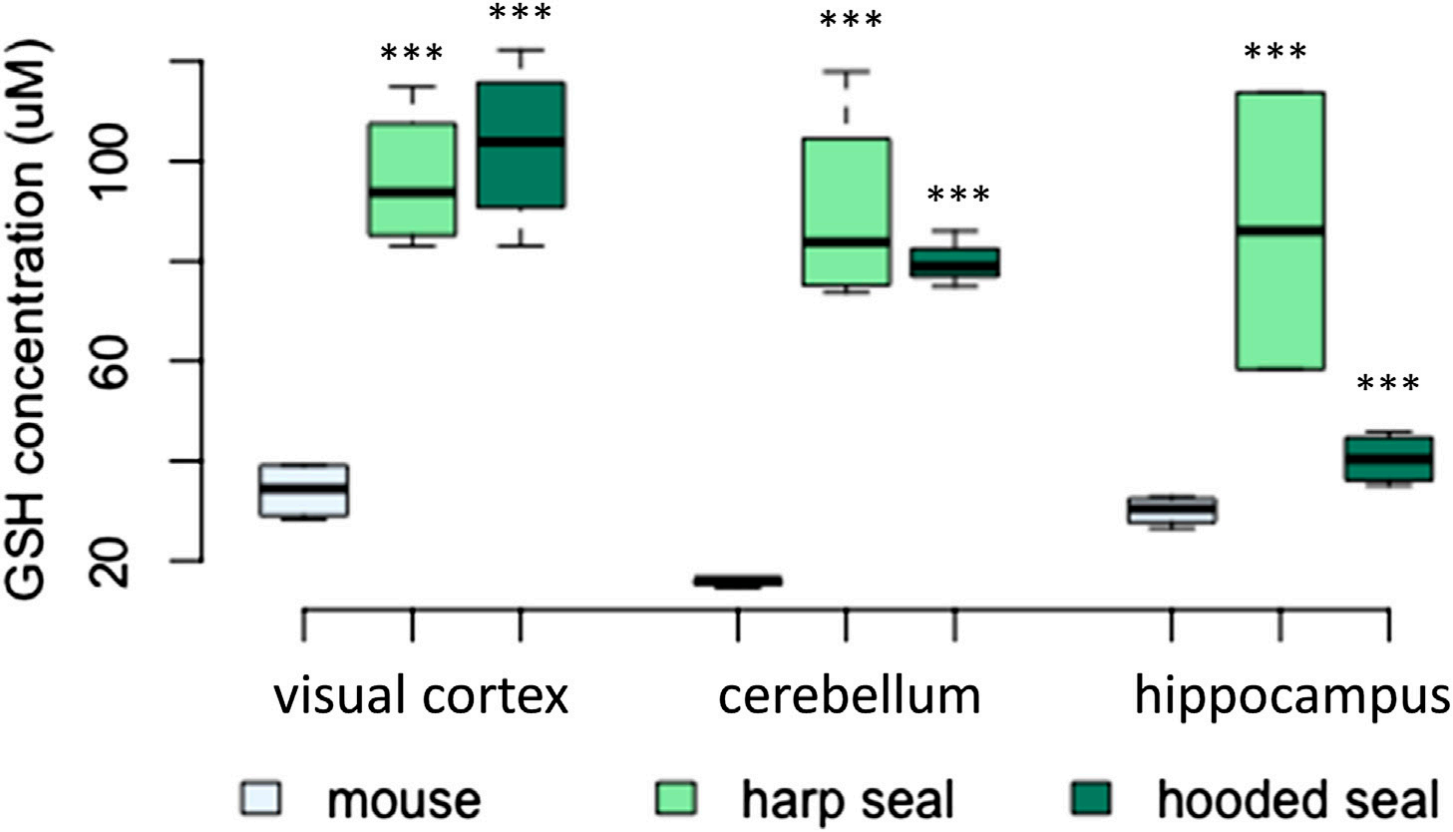
The concentration of reduced glutathione (GSH, μM) in mice, harp seals and hooded seals. Significance levels refer to differences compared to mice in the respective brain region and are represented by asterisks (*p* ≤ 0.05 (*), *p* ≤ 0.01 (**), *p* ≤ 0.01 (***).

As for GSH, both seal species exhibited significantly elevated levels compared to mice (Figure 5; Table 2). The visual cortex had the overall highest GSH concentrations, with 96 ± 7.2 μM (*p* < 0.001) in harp seals and 103 ± 8.3 μM (*p* < 0.001) in hooded seals, compared to only 34 ± 2.9 μM in mice. Further, while harp seals and hooded seals had cerebellar GSH concentrations of 90 ± 10.1 μM (*p* < 0.001) and 80 ± 3.2 μM (*p* < 0.001), respectively, mice had only 16 ± 0.4 μM. Similarly, the hippocampi of harp and hooded seals had GSH levels of 86 ± 17.7 μM (*p* < 0.001) and 100 ± 5.5 μM (*p* < 0.001), while mice had 30 ± 1.5 μM.

### Positive selection

To explore whether positive selection was acting on genes coding for antioxidants in the pinniped lineages, we selected seven pinnipeds and five terrestrial carnivores and fitted two branch-site models (BUSTED and aBSREL). We found no genes for which any positive selection pressure was indicated, for either seal species (Supplementary Table S4).

## Discussion

### Elevated antioxidant gene expression in hooded seal neurons

We here extend the work of Geßner et al. (2022) by investigating the expression levels of all antioxidant genes from the neuron-specific transcriptomes of hooded seals and mice. The majority of differentially expressed genes were more highly expressed in seal neurons (Supplementary Table S2) and thus, our data indicate an overall higher expression of antioxidant genes in hooded seal neurons than in mice, regardless of brain region studied. For example, the S100 calcium-binding protein A9 (S100A9) (Supplmentary Table S2), which plays an important role in the regulation of inflammatory processes and the immune response (Ryckman et al., 2003), was 99.7-fold more highly expressed in the seal compared to the mouse. The expression of S100A9 in neutrophiles and activated macrophages, cells that produce large amounts of ROS during inflammation, suggests that it protects tissues from oxidative damage (Srikrishna, 2012). In murine neutrophils, S100A9 alters mitochondrial homeostasis. Neutrophils lacking S100A9 produce increased levels of mitochondrial O_2_^•−^ when challenged with bacteria (Monteith et al., 2021). In the seal brain, S100A9 may possibly have similar roles in ROS defence and mitochondrial balance, which could explain its high expression in hooded seal neurons.

Another example was selenoprotein T (SELENOT, FC = 17.6, Supplementary Table S2), which possesses a potent oxidoreductase activity and protects dopaminergic neurons in mice from oxidative stress and cell death (Boukhzar et al., 2016).

Two more interesting candidates that were more highly expressed in seal than in mouse neurons, although with less margin, are heme oxygenase 2 (HMOX2, FC = 2.5) and paraoxonase 2 (PON2, FC = 4, Supplementary Table S2). HMOX2 is a constitutively expressed enzyme involved in heme catabolism, by cleaving heme to biliverdin which is then metabolized to bilirubin. Free cellular heme, if not cleaved, can lead to ROS production and membrane lipid peroxidation (Belcher et al., 2010). Both, biliverdin and bilirubin, are potent antioxidants (Barañano et al., 2002). Consequently, HMOX2 activity has an important role in heme homeostasis and cytoprotection. In contrast to the well-studied isoform HMOX1, HMOX2 is more highly expressed in neuronal cells in the forebrain, cerebellum, hippocampus and other brain regions in rats and has functions in cytoprotection and oxygen sensing (Muñoz-Sánchez and Chánez-Cárdenas, 2014). Several studies have shown that HMOX2 gene expression is activated by oxidative stress, while hypoxia can regulate gene expression and translation (Muñoz-Sánchez and Chánez-Cárdenas, 2014 for a review). Interestingly, while HMOX1 expression in skeletal muscle of northern elephant seals is associated with the expression of other antioxidants, correlates with age and was highest in adult females, HMOX2 expression did not vary with age or sex (Piotrowski et al., 2021). Adult females are thought to dive beyond their calculated aerobic dive limit (Hassrick et al., 2007) and thus, the observed elevated expression may be needed to protect them from a more severe risk of oxidative damage (Piotrowski et al., 2021). These results show that the precise interplay of antioxidants may vary with species age and sex.

PON2 is mainly localized in the mitochondria, where it scavenges ROS. Its expression is highest in dopaminergic regions, such as the striata, where it is more highly expressed in astrocytes than in neurons (Costa et al., 2014). PON2 knockdown mice and mice with reduced PON2 levels were more susceptible to oxidative stress than wild type mice (Ng et al., 2006). This indicates that the constitutively high PON2 levels in hooded seals might prevent cellular damage in phases of oxidative stress.

### Antioxidant expression in the cerebellum

While the differential expression found in the neuronal transcriptomes of hooded seals and mice was mostly confirmed using qPCR and enzymatic acitivity assays, the qPCR data of the cerebellum was an exception. For example, SOD1 expression (transcriptome) in hooded seal neurons from the visual cortex was higher than in corresponding cells from mice (Table 1). Concordantly, SOD enzymatic activity was higher in all three brain regions of both seal species than in mice (Figure 2). SOD1 expression (qPCR) confirmed these data in the visual cortex and hippocampus, while the expression of the cerebellum was different across most of the genes studied (Figure 1). Consequently, while activity assays suggested that the cerebellum of seals has an antioxidative capacity similar to other brain regions, the qPCR implies that it was lower. This difference is possibly due to activity assays detecting all isoforms of an enzyme, while the qPCR specifically detects the isoform of interest. Thus, we cannot fully exclude that the cerebellum might have an overall lower antioxidant capacity than the other studied brain regions. Future expression studies could analyse all isoforms of a particular enzyme in the cerebellum and compare a larger number of genes in different brain regions to clarify this observation.

### High SOD levels in the pinniped brain

One of the most common ROS is the highly reactive superoxide anion radical (O_2_^•−^), which is the primary free oxygen radical produced in mitochondria (Murphy, 2009). Superoxide dismutase (SOD) is in the first line of defence against ROS. SOD converts O_2_^•−^ to the more stable hydrogen peroxide (H_2_O_2_) (McCord and Fridovich, 1969). SOD1 was found to be more highly expressed in neurons of the visual cortex in hooded seals than in mouse neurons (Geßner et al., 2022). Even when all transcripts (not only antioxidant genes) were considered, SOD1 was among the top 10 most strongly expressed genes in hooded seal neurons and it was among the top 10 with the highest fold change compared to neurons of mice, which implies its high importance for seal neurons. SOD1 qPCR expression analyses (except for cerebellum) and enzymatic activity assays indicated that constitutively elevated SOD levels might be an important adaptation to diving in pinnipeds and could be relevant in different brain regions.

Mitochondrial energy metabolism is quantitatively the most relevant source of ROS in eukaryotic cells (Kowaltowski et al., 2009). Earlier transcriptome studies of hooded seal neurons and whale brains (whole tissue) have revealed an elevated expression of genes involved in mitochondrial function and oxidative phosphorylation (Krüger et al., 2020; Geßner et al., 2022). However, a study in which only enriched gene ontology terms were considered, but without detailed study of mitochondrial genes, did not find this (Fabrizius et al., 2016). Geßner et al. (2022) suggested that an elevated mitochondrial function, i.e., an elevated aerobic capacity, is important to efficiently use oxygen as far as it is available. However, it might also lead to phases of higher ROS production, especially upon reperfusion as the animal surfaces after a dive, which might necessitate constitutively higher SOD levels in order to prevent ROS leakage from mitochondria.

Our results are in line with previous studies in diving mammals: Blood, heart, kidney and lung tissue of several cetacean, pinniped and manatee species have generally higher SOD activities as compared to domestic pigs and/or other non-diving mammals (Elsner et al., 1998; Filho et al., 2002; Vázquez-Medina et al., 2006). Among diving mammals, SOD activity was positively correlated with dive duration data for the involved species (Righetti et al., 2014), although that does not hold true for all species comparisons (Cantú-Medellín et al., 2011).

### Elevated GPX expression and activity in pinnipeds

After the conversion of O_2_^•−^ by SODs to hydrogen peroxide (H_2_O_2_), H_2_O_2_ can be reduced to water by glutathione peroxidase (GPX) (Lubos et al., 2011). GPX also reduces lipid peroxides and organic hydroperoxides (Esworthy et al., 1991). GPX1, and GPX3 in particular, were more highly expressed in hooded seal neurons compared to mouse neurons. Similar to SOD1, GPX3 was among the top 10 most highly expressed genes and among the top 10 genes with the highest-fold changes compared to neurons of mice, indicating its importance in hooded seal neurons of the visual cortex. Enzymatic activity assays detecting all GPX forms showed increased activity in hooded and harp seals in all brain regions studied. GPX3 expression (qPCR) was only elevated in the visual cortex (and insignificantly increased in the hippocampus) of both seal species compared to mice. Our data suggest that GPX is a relevant component of the antioxidant defence system of diving mammals. Possibly, high conversion rates of O_2_^•–^ to H_2_O_2_ *via* SOD necessitates further processing of H_2_O_2_ by high GPX activity. Our results supplement previous observations of high GPX levels in other tissues than brain, showing that heart, lung and muscle tissue of ringed seals (*Phoca hispida*) has elevated GPX activity compared to domestic pig tissues (*Sus scrofa domesticus*) (Vázquez-Medina et al., 2006). Blood of several cetacean species also showed elevated GPX activities compared to terrestrial mammals (Filho et al., 2002). Similar to SOD, some authors found higher GPX activities in blood from species with longer submergence times (Righetti et al., 2014), while other studies did not find such a correlation when heart, brain, lung, kidney and muscle tissues were analysed (Cantú-Medellín et al., 2011).

The conversion of H_2_O_2_ to water by GPX goes along with the oxidation of the reduced glutathione (GSH) to glutathione disulphide (GSSG) (Lauterburg et al., 1984). The glutathione system, thus, plays a central role in antioxidant defence. For that reason, we studied it in greater detail.

### The glutathione system: GSH and GSR levels, and GSH biosynthesis

GSH is a non-enzymatic antioxidant that functions as scavenger of free radicals (e.g. Jimenez and Speisky, 2000), as a substrate in GPX reactions, in reactions catalyzed by glutathione-S-transferases (GST) possessing peroxidase function and by phospholipide hydroperoxide glutathione peroxidase, and in reactions with *α*-tocopherol (vitamin E) protecting lipids from ROS damage (Meister, 1983). All these processes lead to the oxidation of GSH to glutathione disulfide (GSSG). Consequently, the organism depends on a GSH concentration that is sufficient to facilitate these reactions. We expected elevated GSH levels in the pinniped brain compared to mice and our data confirmed this hypothesis. Our results are in line with elevated GSH levels in several tissues of ringed seals compared to domestic pigs (Vázquez-Medina et al., 2007) and in blood from several cetaceans and one semiaquatic species (neotropical otter (*Lontra longicaudis annectens*)) compared to terrestrial mammals (Filho et al., 2002; García-Castañeda et al., 2017).

We were unable to correctly measure total glutathione levels (GSH + GSSG) and thus, could not calculate the GSH/GSSG ratio in spite of using two different assays, varying preparation of samples, and testing different individuals of seals and mice. In mice, the expected GSSG concentration should be ∼0.7% of the GSH concentration, as was found in the cerebral cortex, cerebellum and brain stem in mice (Folbergrová et al., 1979). Filho et al. (2002) found no difference in blood total glutathione content (ratio was not calculated) between cetaceans and terrestrial species. García-Castañeda et al. (2017) calculated the GSSG/total glutathione ratio, which was lower in diving species than in non-diving mammals, ascribing diving species a higher capacity for GSH-dependent reactions.

Often, the GSH/GSSG ratio is considered an indicator of the redox state and, thus, the wellbeing of a cell. Drastic changes in this ratio indicate that there is an imbalance in the redox metabolism. However, enzymatic reactions involving GSH depend on the GSH-concentration, not on GSSG, as predicted by the Nernst equation, and are typically not affected by GSSG (Flohé, 2013). Even though our dataset is partially incomplete, given the missing data on total glutathione levels, this missing value might not compromise our overall findings.

**FIGURE 6:**
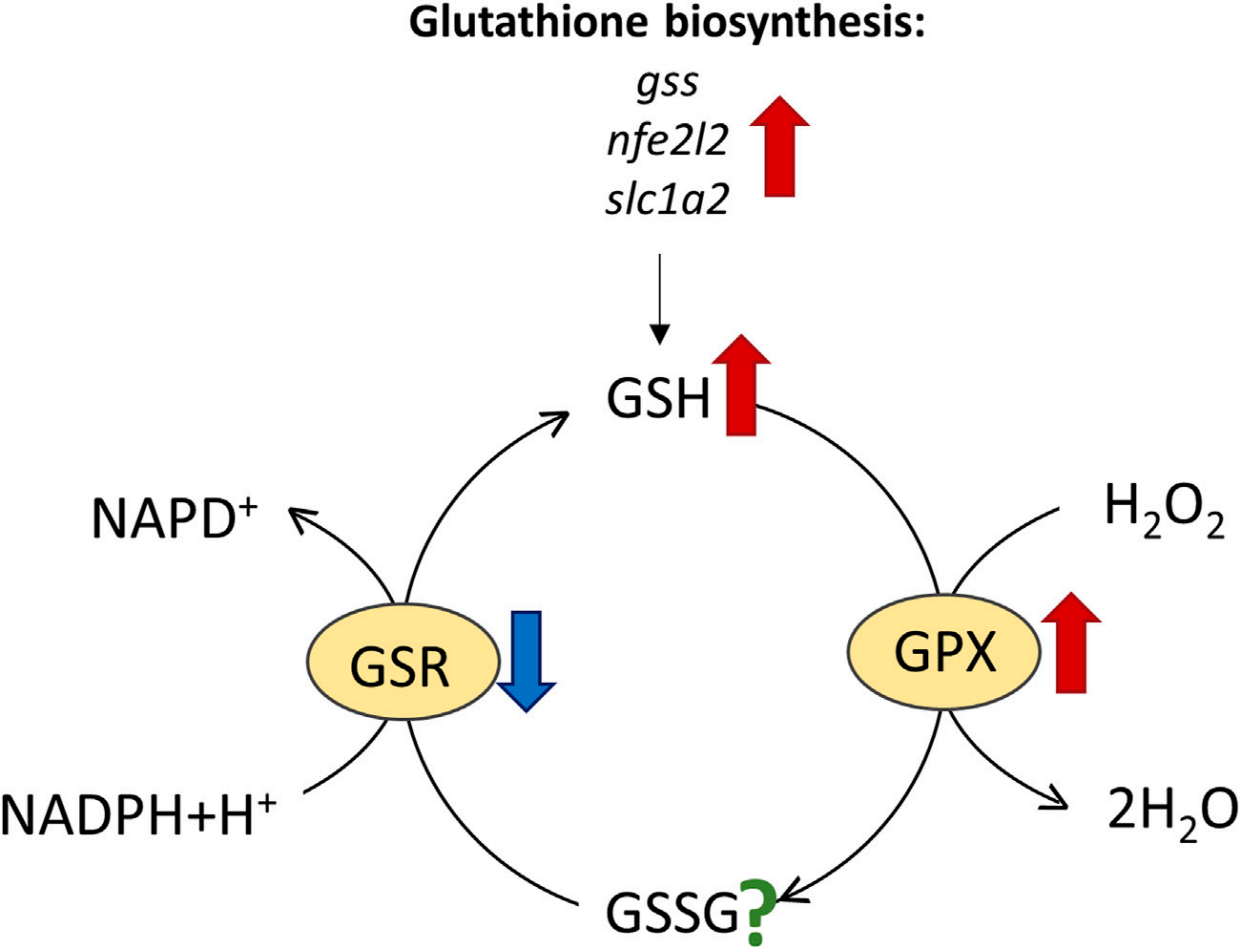
Reactions involving reduced glutathione (GSH), such as the conversion of H_2_O_2_ to water by glutathione peroxidase (GPX), oxidize GSH to glutathione disulphide (GSSG), which is then re-cycled to GSH by glutathione reductase (GSR). Results of elevated expression/enzymatic activity in harp and/or hooded seals vs. mice are indicated by red arrows and reduced activity by blue arrows, while the question mark signifies unknown activity change.

Glutathione reductase (GSR) mediates the transition of GSSG to GSH that is necessary for the recovery of the GSH pool (Figure 6). Since GSH levels were high in seals, we expected similarly elevated GSR levels. GSR expression was only moderately, but yet significantly, elevated in hooded seal neurons. However, the GSR activity was found to be similar, or even lower, in seals than in mice. This is surprising, since blood samples from cetaceans showed elevated GSR activities compared with terrestrial mammals (Filho et al., 2002). Blood from seals and brain tissue from mice can be sampled easier and faster than brain tissue of seals. Given that GSR gene expression was high and that enzymes differ in stability, we cannot exclude that GSR possibly degraded during the 10 min it took to sample and preserve seal brain tissues. However, since GSR activity increases in response to rising GSSG levels during oxidative stress, it is possible that significant increases in GSR activity are a characteristic of tissue in acute oxidative stress rather than a constitutive measure (Jones, 2002).

Apart from GSR, other enzymes and factors also contribute to the GSH level. Since GST conjugates GSH to electrophilic compounds, a reduced activity of GSTs - as found in pinnipeds-might draw relatively less GSH from the GSH pool, although to the best of our knowledge we do not know the effect size of GST activity on GSH concentration. In hooded seal neurons, glutathione synthase (GSS, EC 6.3.2.3), nuclear factor erythroid 2-related Factor 2 (NFE2L2), a transcription factor activating glutathione biosynthesis and the expression of other antioxidants (Cullinan and Diehl, 2004; Eggler et al., 2009), and solute carrier family 1 member 2 (SLC1A2, EAAT2), were all more highly expressed than in mice (Supplementary Table 2). SLC1A2 is a membrane-bound transporter mediating neuronal uptake of amino-acids, which, among other things, is responsible for clearing glutamate from the synaptic cleft (e.g., Arriza et al., 1994). Additionally, mouse cortical neuron culture studies show that SLC1A2 and SLC1A3 also facilitate the uptake of cysteine, which is a rate-limiting factor in glutathione synthesis (Chen and Swanson, 2003). Taken together, our data suggest that elevated levels of GSH in the seal brain are, at least in part, explained by elevated GSH biosynthesis.

### High expression of specific GSTs, but overall reduced GST activity in the pinniped brain

The glutathione-S-transferases (GSTs) are enzymes that conjugate GSH to electrophilic reactive compounds that would otherwise bind to proteins or nucleic acid, leading to cellular damage. Some GSTs are also able to detoxify hydroperoxides (Cooper and Kristal, 1997; Sherratt and Hayes, 2001) and certain cytosolic GSTs also catalyse GSH-dependent reduction of lipid peroxides (Cooper and Kristal, 1997). We found GSTK1, GSTO1 and GSTO2 to have a noticeably elevated expression in hooded seal neurons compared to mice (Table 1). We selected GSTK1 and GSTO1 for qPCR analysis that confirmed a higher pinniped expression in the brain regions studied (except for the cerebellum, Figure 1). Surprisingly, the GST assay, detecting all GSTs, showed a clearly *lower* GST activity in both seal species. We therefore extracted gene expression values for all GSTs present in our neuronal transcriptome (Supplementary Table S5) and found that other GSTs, such as GSTM1 and GSTM3, are markedly less expressed in neurons of the hooded seal than in mice, which caused the overall GST expression to be similar between the species. The broad and partly overlapping substrate specificity of GSTs (Wätjen and Fritsche, 2010) makes it difficult to explain why some GSTs might be more, or less, expressed in the seal brain. However, these data suggest that GSTs in general might not be a crucial component in the antioxidant defence system of the seal brain.

Previous studies on GST activities in tissues from diving mammals also reveal mixed results. GST activities were higher in the blood of southern elephant seals (*Mirounga leonina*), marine manatee (*Trichechus manatus*) and 3 dolphin species, when compared to terrestrial mammals (Filho et al., 2002). In ringed seal tissues, GST activity was higher and lower in heart and liver, respectively, compared to domestic pigs, but similar in lung, kidney and muscle (Vázquez-Medina et al., 2006). GST activity does not appear to correlate positively with diving capacity/behaviour, since GST activities of short-duration/shallow divers *versus* deep/long-duration divers were similar in seven tissues studied (Cantú-Medellín et al., 2011). Only in blood, GST activity appears to increase with diving ability in seals (Righetti et al., 2014). Interestingly, cetaceans have undergone a reduction of the GST gene family, with bowhead whales (*Balaena mysticetus*) having 16 GSTs, while mice have 30 copies and Weddell seals have an intermediate number of 22 GSTs (Tian et al., 2019). The hypoxia tolerant naked mole rat (*Heterocephalus glaber*) has a high number of the cytosolic mu subclass of GSTs responsible for cellular detoxification (10 copies). Further, some GSTs have signatures of positive selection (GSTP2) in five mammalian lineages, while others (GSTP1) are conserved across mammals (Tian et al., 2019). We did not find GSTO1 to be positively selected in seals. To summarize, a detailed study of the orchestra of expansion/reduction, expression and functional changes of certain GST subclasses, rather than a measurement of the overall GST activity alone, would better explain the adaptation to differently adverse environments.

### The glutaredoxin and thioredoxin systems

In mammalian cells, the cytosolic and mitochondrial thioredoxin systems and the glutathione-glutaredoxin system have a key role in antioxidant defence and have a great impact on the cellular redox state (Lu and Holmgren, 2014). We here examine a representative of each system.

### Mixed GLRX2 expression in the pínniped brain

Glutaredoxin 2 (GLRX2) is an antioxidant enzyme belonging to the glutaredoxin family, which consists of small redox proteins of the thioredoxin superfamily. It catalyses the transfer of electrons from GSH to disulfides (Holmgren, 1989), which maintains the intracellular redox homeostasis in the face of oxidative stress (Jung and Thomas, 1996). GLRX2 is expressed in a range of tissues, including neurons of the mammalian brain (e.g., Padilla et al., 1992; Garcia-Pardo et al., 1999; Karunakaran et al., 2007; Mailloux et al., 2014; Upadhyaya et al., 2015). GLRX2 is an interesting candidate when studying the antioxidant defence of diving mammals since it protects mouse cardiomyocytes from hypoxia-/reoxygenation-induced oxidative stress, apoptosis and inflammation (Li et al., 2021). GLRX2 facilitates mitochondrial redox homeostasis and thus, contributes to the functional integrity of mitochondria (Karunakaran et al., 2007). Since whale brains (Krüger et al., 2020) and hooded seal neurons (Geßner et al., 2022) appear to have a high capacity for oxidative phosphorylation and possibly an abundant number of mitochondria based on a high expression of components of the mitochondrial envelope in hooded seal neurons (but see Mitz et al., 2009), we could perhaps have anticipated an elevated expression of GLRX2. Instead, we found GLRX2 to be less expressed in hooded seal neurons than in mice and tested whether this trend is true for other brain regions, as well. Indeed, GLRX2 expression in harp seals and hooded seals was lower or similar compared to mice. Further, GLRX and GLRX3 were also less expressed in hooded seal neurons than in mice (Supplementary Table S2).

Since other essential components of the antioxidant system, such as SOD and GPX, appear to be constitutively more highly expressed in seals, it might not be necessary for the glutaredoxin system to be constitutively more active. Instead, upregulation of GLRX2 might be triggered by oxidative stress, as observed in mouse cardiomyocytes upon hypoxia/reoxygenation treatment (Li et al., 2021). Further support comes from diving-induced upregulation of GLRX2 in the blood of bottlenose dolphins (Blawas et al., 2021).

### TXNRD3 expression was higher (harp seal) or similar (hooded seal) to mice

Thioredoxin reductase 3 (TXNRD3) is a representative of the thioredoxin system. Among the three thioredoxin reductase isoenzymes known in mammals, TXNRD3 is the only one that contains an additional N-terminal glutaredoxin domain, which enables this isoenzyme to be involved in both the thioredoxin and the glutaredoxin systems (Arnér, 2009). TXNRD3 reduces thioredoxin. The thioredoxin system provides electrons to thiol-dependent peroxidases, to detoxify reactive oxygen and nitrogen species (Lu and Holmgren, 2014). TXNRD1 and TXNRD3 were both more highly expressed in hooded seal neurons compared to mice (Supplementary Table S2), while thioredoxin was not differentially expressed (data not shown). Expression data in other brain regions were mixed, with TXNRD3 being more highly expressed in harp seals (except for cerebellum), while being similarly expressed in hooded seals compared to mice. To the best of our knowledge, the thioredoxin system has not been studied in hypoxia-tolerant species. However, Alzheimer’s disease (AD) is characterized by hypoxia and oxidative damage and - similar to our findings–increased TXNRD levels, but decreased thioredoxin levels were found in AD brains. The increased TXNRD levels alone were interpreted as insufficiently protective (Lovell et al., 2000). Similar to the glutaredoxin system, thioredoxins may, thus, not belong to the constitutively increased antioxidative defence system of the pinniped brain.

### Antioxidant genes appear to be conserved in pinnipeds

We found that none of the here studied antioxidant genes (GPX3, SOD1, GSTK1 GSTO1, TXNRD3, GLRX2 and GSR) have been subject to positive selection in pinnipeds when compared to non-diving carnivores (Supplementary Table S4). Since we found elevated expression and enzymatic activity levels in several of these antioxidants, the results suggest that selection might have favoured increased levels, whereas gene sequence and function were conserved in pinnipeds. There is, however, evidence that positive selection is relevant in the adaptation to the aquatic life in general and to hypoxia in particular. For instance, genes for oxygen transport (hemoglobin-α and -β, myoglobin) and genes regulating vasoconstriction show positive selection in cetaceans (Tian et al., 2016). Further, GSR was positively selected in the bottlenose dolphin (*Tursiops truncatus*) and both GSR and GPX2 show cetacean-specific amino-acid substitution (Yim et al., 2014). Interestingly, there are three pinniped-specific amino-acid changes in GSR (Supplementary Figure S1) and BUSTED found evidence for diversifying selection, i.e., at least one site in at least one branch has undergone positive selection. However, aBSREL failed to identify one or more branches. Consequently, GSR might not truly be positively selected in pinnipeds, but it might be carefully interpreted as weak signals of positive selection that are not beyond the threshold of being clearly characterized as positive selection.

In this study, pinniped antioxidant capacity was compared to that of mice. While mice are a well-accepted organism for scientific purposes with clear advantages in availability and handling, it would be ideal to compare pinnipeds to non-diving mammals of similar body size, since metabolic rate correlates with body mass (e.g., Gillooly et al., 2001) which might also affect ROS production and, thus, antioxidant capacity. Previous studies compared antioxidants in ringed seals with domestic pigs and found a similar trend as in this study, i.e., a generally elevated antioxidant capacity in ringed seals.

## Conclusion

We conclude that the brains of harp and hooded seals have an overall constitutively enhanced antioxidant defence system, as has been generally found in other tissues of diving mammals. We found that not the antioxidant system as a whole, but some of its essential components, such as SOD and GPX, were constitutively elevated, whereas others, like the glutaredoxin and thioredoxin systems, were not enhanced (Table 2 for a summary). These systems and possibly other antioxidants are likely activated as needed, and may be further boosted by diving-induced mechanisms, as observed for GLRX2 in bottlenose dolphins (Blawas et al., 2021). Since we studied two pinniped species, our findings might generally be similar in other pinnipeds. However, the precise orchestra of protective mechanisms, including antioxidant capacity, might vary with species based on their life history traits, with age and with sex. For instance, the muscle expression of several antioxidant genes increased with age and diving ability in northern elephant seals and hooded seals (Vázquez-Medina et al., 2011; Piotrowski et al., 2021) and some antioxidants were observed to be higher in northern elephant females than in males (Piotrowski et al., 2021).

## Data Availability

The original contributions presented in the study are included in the article/Supplementary Material, further inquiries can be directed to the corresponding author.

## References

[B1] Arnér E. S. (2009). Focus on mammalian thioredoxin reductases—Important selenoproteins with versatile functions. Biochim. Biophys. Acta 1790, 495–526. 10.1016/j.bbagen.2009.01.014 19364476

[B2] Arriza J. L. Fairman W. A. Wadiche J. I. Murdoch G. H. Kavanaugh M. P. Amara S. G. (1994). Functional comparisons of three glutamate transporter subtypes cloned from human motor cortex. J. Neurosci 14, 5559–5569. 10.1523/JNEUROSCI.14-09-05559.1994 7521911 PMC6577102

[B3] Barañano D. E. Rao M. Ferris C. D. Snyder S. H. (2002). Biliverdin reductase: A major physiologic cytoprotectant. Proc. Natl. Acad. Sci. U. S. A 99, 16093–16098. 10.1073/pnas.252626999 12456881 PMC138570

[B4] Belcher J. D. Beckman J. D. Balla G. Balla J. Vercellotti G. (2010). Heme degradation and vascular injury. Antioxid. Redox Signal 12, 233–248. 10.1089/ars.2009.2822 19697995 PMC2821146

[B5] Benjamini Y. Hochberg Y. (1995). Controlling the false discovery rate: A practical and powerful approach to multiple testing. J. R. Stat. Soc. Ser. B Methodol 57, 289–300. 10.1111/j.2517-6161.1995.tb02031.x

[B6] Blawas A. M. Ware K. E. Schmaltz E. Zheng L. Spruance J. Allen A. S. (2021). An integrated comparative physiology and molecular approach pinpoints mediators of breath-hold capacity in dolphins. Evol. Med. Public Health 9, 420–430. 10.1093/emph/eoab036 35169481 PMC8833867

[B7] Blix A. S. (2018). Adaptations to deep and prolonged diving in phocid seals. J. Exp. Biol 221, jeb182972. 10.1242/jeb.182972 29934417

[B8] Boukhzar L. Hamieh A. Cartier D. Tanguy Y. Alsharif I. Castex M. (2016). Selenoprotein T exerts an essential oxidoreductase activity that protects dopaminergic neurons in mouse models of Parkinson's disease. Antioxid. Redox Signal 24, 557–574. 10.1089/ars.2015.6478 26866473 PMC4840926

[B9] Cantú-Medellín N. Byrd B. Hohn A. Vázquez-Medina J. P. Zenteno-Savín T. (2011). Differential antioxidant protection in tissues from marine mammals with distinct diving capacities. Shallow/short vs. deep/long divers. Comp. Biochem. Physiol. A Mol. Integr. Physiol 158, 438–443. 10.1016/j.cbpa.2010.11.029 21147244

[B10] Chen Y. Swanson R. A. (2003). The glutamate transporters EAAT2 and EAAT3 mediate cysteine uptake in cortical neuron cultures. J. Neurochem 84, 1332–1339. 10.1046/j.1471-4159.2003.01630.x 12614333

[B11] Cooper A. J. Kristal B. S. (1997). Multiple roles of glutathione in the central nervous system. Biol. Chem 378, 793–802. 9377474

[B12] Costa L. G. de Laat R. Dao K. Pellacani C. Cole T. B. Furlong C. E. (2014). Paraoxonase-2 (PON2) in brain and its potential role in neuroprotection. Neurotoxicology 43, 3–9. 10.1016/j.neuro.2013.08.011 24012887 PMC3942372

[B13] Cullinan S. B. Diehl J. A. (2004). PERK-dependent activation of Nrf2 contributes to redox homeostasis and cell survival following endoplasmic reticulum stress. J. Biol. Chem 279, 20108–20117. 10.1074/jbc.M314219200 14978030

[B14] Duan W. Zhang Y.-P. Hou Z. Huang C. Zhu H. Zhang C.-Q. (2016). Novel insights into NeuN: From neuronal marker to splicing regulator. Mol. Neurobiol 53, 1637–1647. 10.1007/s12035-015-9122-5 25680637

[B15] Eggler A. L. Small E. Hannink M. Mesecar A. D. (2009). Cul3-mediated Nrf2 ubiquitination and antioxidant response element (ARE) activation are dependent on the partial molar volume at position 151 of Keap1. Biochem. J 422, 171–180. 10.1042/BJ20090471 19489739 PMC3865926

[B16] Elsner R. Øyasæter S. Almaas R. Saugstad O. D. (1998). Diving seals, ischemia-reperfusion and oxygen radicals. Comp. Biochem. Physiol. A Mol. Integr. Physiol 119, 975–980. 10.1016/s1095-6433(98)00012-9 9773490

[B17] Esworthy R. S. Chu F.-F. Akman S. Doroshow J. H. Paxton R. J. (1991). Characterization and partial amino acid sequence of human plasma glutathione peroxidase. Arch. Biochem. Biophys 286, 330–336. 10.1016/0003-9861(91)90048-n 1897960

[B18] Fabrizius A. Hoff M. L. M. Engler G. Folkow L. P. Burmester T. (2016). When the brain goes diving: Transcriptome analysis reveals a reduced aerobic energy metabolism and increased stress proteins in the seal brain. BMC genomics 17, 1–11. 10.1186/s12864-016-2892-y 27507242 PMC4979143

[B19] Filho D. Sell F. Ribeiro L. Ghislandi M. Carrasquedo F. Fraga C. (2002). Comparison between the antioxidant status of terrestrial and diving mammals. Comp. Biochem. Physiol. A Mol. Integr. Physiol 133, 885–892. 10.1016/s1095-6433(02)00253-2 12443944

[B20] Flohé L. (2013). The fairytale of the GSSG/GSH redox potential. Biochim. Biophys. Acta 1830, 3139–3142. 10.1016/j.bbagen.2012.10.020 23127894

[B21] Folbergrová J. Rehncrona S. Siesjö B. K. (1979). Oxidized and reduced glutathione in the rat brain under normoxic and hypoxic conditions. J. Neurochem 32, 1621–1627. 10.1111/j.1471-4159.1979.tb02271.x 36446

[B22] Fox J. Bouchet-Valat M. Andronic L. Ash M. Boye T. Calza S. 2019. Package ‘Rcmdr’.

[B23] Fridovich I. (1998). Oxygen toxicity: A radical explanation. J. Exp. Biol 201, 1203–1209. 10.1242/jeb.201.8.1203 9510531

[B24] Garcia-Pardo L. Granados M. Gaytan F. Padilla C. Martinez-Galisteo E. Morales C. (1999). Immunolocalization of glutaredoxin in the human corpus luteum. Mol. Hum. Reprod 5, 914–919. 10.1093/molehr/5.10.914 10508218

[B25] García Castañeda O. Gaxiola‐Robles R. Kanatous S. Zenteno‐Savín T. (2017). Circulating glutathione concentrations in marine, semiaquatic, and terrestrial mammals. Mar. Mamm. Sci 33, 738–747. 10.1111/mms.12391

[B26] Gelain D. P. Dalmolin R. J. Belau V. L. Moreira J. C. Klamt F. Castro M. (2009). A systematic review of human antioxidant genes. Front. Biosci 14, 4457–4463. 10.2741/3541 19273363

[B27] Geßner C. Krüger A. Folkow L. P. Fehrle W. Mikkelsen B. Burmester T. (2022). Transcriptomes suggest that pinniped and cetacean brains have a high capacity for aerobic metabolism while reducing energy-intensive processes such as synaptic transmission. Front. Mol. Neurosci 15, 877349. 10.3389/fnmol.2022.877349 35615068 PMC9126210

[B28] Gillooly J. F. Brown J. H. West G. B. Savage V. M. Charnov E. L. (2001). Effects of size and temperature on metabolic rate. Science 293, 2248–2251. 10.1126/science.1061967 11567137

[B29] Halliwell B. Gutteridge J. M. (2015). Free radicals in biology and medicine. Oxford, USA: Oxford University Press.

[B30] Hassrick J. L. Crocker D. E. Zeno R. L. Blackwell S. B. Costa D. P. Le Boeuf B. J. (2007). Swimming speed and foraging strategies of northern elephant seals. Deep Sea Res. Part II Top. Stud. Oceanogr 54, 369–383. 10.1016/j.dsr2.2006.12.001

[B31] Hermes-Lima M. Moreira D. C. Rivera-Ingraham G. A. Giraud-Billoud M. Genaro-Mattos T. C. Campos É. G. (2015). Preparation for oxidative stress under hypoxia and metabolic depression: Revisiting the proposal two decades later. Free Radic. Biol. Med 89, 1122–1143. 10.1016/j.freeradbiomed.2015.07.156 26408245

[B32] Holmgren A. (1989). Thioredoxin and glutaredoxin systems. J. Biol. Chem 264, 13963–13966. 10.1016/s0021-9258(18)71625-6 2668278

[B33] Jeong Y. J. Son Y. Han N.-K. Choi H.-D. Pack J.-K. Kim N. (2018). Impact of long-term RF-EMF on oxidative stress and neuroinflammation in aging brains of C57BL/6 mice. Int. J. Mol. Sci 19, 2103. 10.3390/ijms19072103 30029554 PMC6073444

[B34] Jimenez I. Speisky H. (2000). Effects of copper ions on the free radical-scavenging properties of reduced gluthathione: Implications of a complex formation. J. Trace Elem. Med. Biol 14, 161–167. 10.1016/S0946-672X(00)80005-X 11130853

[B35] Jones D. P. (2002).Redox potential of GSH/GSSG couple: Assay and biological significance, Methods Enzym, 348. 93–112. 10.1016/s0076-6879(02)48630-2 11885298

[B36] Jung C.-H. Thomas J. A. (1996). S-glutathiolated hepatocyte proteins and insulin disulfides as substrates for reduction by glutaredoxin, thioredoxin, protein disulfide isomerase, and glutathione. Arch. Biochem. Biophys 335, 61–72. 10.1006/abbi.1996.0482 8914835

[B37] Karunakaran S. Saeed U. Ramakrishnan S. Koumar R. C. Ravindranath V. (2007). Constitutive expression and functional characterization of mitochondrial glutaredoxin (Grx2) in mouse and human brain. Brain Res 1185, 8–17. 10.1016/j.brainres.2007.09.019 17961515

[B38] Kassambara A. (2020). Ggpubr:“ggplot2” based publication ready plots (R package version. [Computer software].https://CRAN.R.project.org/package=ggpubr.ggpubr:‘ggplot2’based.publication.ready.plots.R.Package.Version.0.4.0.

[B39] Kassambara A. (2021). Rstatix: Pipe-friendly framework for basic statistical tests. https://CRAN.R.project.org/package=rstatix.R.package.version.0.7.0.

[B40] Katoh K. Standley D. M. (2013). MAFFT multiple sequence alignment software version 7: Improvements in performance and usability. Mol. Biol. Evol 30, 772–780. 10.1093/molbev/mst010 23329690 PMC3603318

[B41] Kevin L. G. Novalija E. Stowe D. F. (2005). Reactive oxygen species as mediators of cardiac injury and protection: The relevance to anesthesia practice. Anesth. Analg 101, 1275–1287. 10.1213/01.ANE.0000180999.81013.D0 16243980

[B42] Kowaltowski A. J. de Souza-Pinto N. C. Castilho R. F. Vercesi A. E. (2009). Mitochondria and reactive oxygen species. Free Radic. Biol. Med 47, 333–343. 10.1016/j.freeradbiomed.2009.05.004 19427899

[B43] Krüger A. Fabrizius A. Mikkelsen B. Siebert U. Folkow L. P. Burmester T. (2020). Transcriptome analysis reveals a high aerobic capacity in the whale brain. Comp. Biochem. Physiol. A Mol. Integr. Physiol 240, 110593. 10.1016/j.cbpa.2019.110593 31676411

[B44] Kumral A. Tugyan K. Gonenc S. Genc K. Genc S. Sonmez U. (2005). Protective effects of erythropoietin against ethanol-induced apoptotic neurodegenaration and oxidative stress in the developing C57BL/6 mouse brain. Brain Res. Dev. Brain Res 160, 146–156. 10.1016/j.devbrainres.2005.08.006 16236368

[B45] Lau M. K. (2013). Dtk: Dunnett-Tukey-Kramer pairwise multiple comparison test adjusted for unequal variances and unequal sample sizes. R. package version 3.

[B46] Lauterburg B. H. Smith C. V. Hughes H. Mitchell J. (1984). Biliary excretion of glutathione and glutathione disulfide in the rat. Regulation and response to oxidative stress. J. Clin. Investig 73, 124–133. 10.1172/JCI111182 6690473 PMC424981

[B47] Li C. Xin H. Shi Y. Mu J. (2021). Glutaredoxin 2 protects cardiomyocytes from hypoxia/reoxygenation-induced injury by suppressing apoptosis, oxidative stress, and inflammation via enhancing Nrf2 signaling. Int. Immunopharmacol 94, 107428. 10.1016/j.intimp.2021.107428 33581580

[B48] Lovell M. A. Xie C. Gabbita S. P. Markesbery W. R. (2000). Decreased thioredoxin and increased thioredoxin reductase levels in Alzheimer’s disease brain. Free Radic. Biol. Med 28, 418–427. 10.1016/s0891-5849(99)00258-0 10699754

[B49] Lu J. Holmgren A. (2014). The thioredoxin antioxidant system. Free Radic. Biol. Med 66, 75–87. 10.1016/j.freeradbiomed.2013.07.036 23899494

[B50] Lubos E. Loscalzo J. Handy D. E. (2011). Glutathione peroxidase-1 in health and disease: From molecular mechanisms to therapeutic opportunities. Antioxid. Redox Signal 15 (7), 1957–1997. 10.1089/ars.2010.3586 21087145 PMC3159114

[B51] Mailloux R. J. Xuan J. Y. McBride S. Maharsy W. Thorn S. Holterman C. E. (2014). Glutaredoxin-2 is required to control oxidative phosphorylation in cardiac muscle by mediating deglutathionylation reactions. J. Biol. Chem 289, 14812–14828. 10.1074/jbc.M114.550574 24727547 PMC4031535

[B52] McCord J. M. Fridovich I. (1969). Superoxide dismutase: An enzymic function for erythrocuprein (hemocuprein). J. Biol. Chem 244, 6049–6055. 10.1016/s0021-9258(18)63504-5 5389100

[B53] Meir J. U. Champagne C. D. Costa D. P. Williams C. L. Ponganis P. J. (2009). Extreme hypoxemic tolerance and blood oxygen depletion in diving elephant seals. Am. J. Physiol. Regul. Integr. Comp. Physiol 297, R927–R939. 10.1152/ajpregu.00247.2009 19641132

[B54] Meister A. (1983). Selective modification of glutathione metabolism. Science 220, 472–477. 10.1126/science.6836290 6836290

[B55] Milkovic L. Cipak Gasparovic A. Cindric M. Mouthuy P.-A. Zarkovic N. (2019). Short overview of ROS as cell function regulators and their implications in therapy concepts. Cells 8, 793. 10.3390/cells8080793 31366062 PMC6721558

[B56] Mitz S. Reuss S. Folkow L. Blix A. Ramirez J.-M. Hankeln T. (2009). When the brain goes diving: Glial oxidative metabolism may confer hypoxia tolerance to the seal brain. Neuroscience 163, 552–560. 10.1016/j.neuroscience.2009.06.058 19576963

[B57] Monteith A. J. Miller J. M. Maxwell C. N. Chazin W. J. Skaar E. P. (2021). Neutrophil extracellular traps enhance macrophage killing of bacterial pathogens. Sci. Adv 7, eabj2101. 10.1126/sciadv.abj2101 34516771 PMC8442908

[B58] Muñoz-Sánchez J. Chánez-Cárdenas M. E. (2014). A review on hemeoxygenase-2: Focus on cellular protection and oxygen response. Oxid. Med. Cell. Longev 2014, 604981. 10.1155/2014/604981 25136403 PMC4127239

[B59] Murphy M. P. (2009). How mitochondria produce reactive oxygen species. Biochem. J 417, 1–13. 10.1042/BJ20081386 19061483 PMC2605959

[B60] Murrell B. Weaver S. Smith M. D. Wertheim J. O. Murrell S. Aylward A. (2015). Gene-wide identification of episodic selection. Mol. Biol. Evol 32, 1365–1371. 10.1093/molbev/msv035 25701167 PMC4408417

[B61] Ng C. J. Hama S. Y. Bourquard N. Navab M. Reddy S. T. (2006). Adenovirus mediated expression of human paraoxonase 2 protects against the development of atherosclerosis in apolipoprotein E-deficient mice. Mol. Genet. Metab 89, 368–373. 10.1016/j.ymgme.2006.07.004 16935014

[B62] Padilla C. A. Martínez-Galisteo E. López-Barea J. Holmgren A. Bárcena J. A. (1992). Immunolocalization of thioredoxin and glutaredoxin in mammalian hypophysis. Mol. Cell. Endocrinol 85, 1–12. 10.1016/0303-7207(92)90119-q 1526311

[B63] Piotrowski E. R. Tift M. S. Crocker D. E. Pearson A. B. Vázquez-Medina J. P. Keith A. D. (2021). Ontogeny of carbon monoxide-related gene expression in a deep-diving marine mammal. Front. Physiol 12, 1841. 10.3389/fphys.2021.762102 PMC8567018 34744798

[B64] Pond S. L. K. Frost S. D. (2005). Datamonkey: Rapid detection of selective pressure on individual sites of codon alignments. Bioinformatics 21, 2531–2533. 10.1093/bioinformatics/bti320 15713735

[B65] Ponganis P. J. (2011). Diving mammals. Compr. Physiol 1, 447–465. 10.1002/cphy.c091003 23737181

[B66] Qvist J. Hill R. D. Schneider R. C. Falke K. J. Liggins G. C. Guppy M. (1986). Hemoglobin concentrations and blood gas tensions of free-diving Weddell seals. J. Appl. Physiol 61, 1560–1569. 10.1152/jappl.1986.61.4.1560 3096941

[B67] R Core Team (2013). R: A language and environment for statistical computing. https://www.yumpu.com/en/document/view/6853895/r-a-language-and-environment-for-statistical-computing.

[B68] Ray P. D. Huang B.-W. Tsuji Y. (2012). Reactive oxygen species (ROS) homeostasis and redox regulation in cellular signaling. Cell. Signal 24, 981–990. 10.1016/j.cellsig.2012.01.008 22286106 PMC3454471

[B69] Righetti B. Simões-Lopes P. Uhart M. Wilhelm Filho D. (2014). Relating diving behavior and antioxidant status: Insights from oxidative stress biomarkers in the blood of two distinct divers, *Mirounga leonina* and arctocephalus australis. Comp. Biochem. Physiol. A Mol. Integr. Physiol 173, 1–6. 10.1016/j.cbpa.2014.02.017 24607367

[B70] Ryckman C. Vandal K. Rouleau P. Talbot M. Tessier P. A. (2003). Proinflammatory activities of S100: Proteins S100A8, S100A9, and S100a8/A9 induce neutrophil chemotaxis and adhesion. J. Immunol 170, 3233–3242. 10.4049/jimmunol.170.6.3233 12626582

[B71] Scholander P. F. (1940). Experimental Investigations On The Respiratory Function In Diving Mammals And Birds. I kommisjon hos Jacob Dybwad. Oslo, Norway,

[B72] Sheldon R. A. Sedik C. Ferriero D. M. (1998). Strain-related brain injury in neonatal mice subjected to hypoxia–ischemia. Brain Res 810, 114–122. 10.1016/s0006-8993(98)00892-0 9813271

[B73] Sherratt P. J. Hayes J. D. (2001). Glutathione S-transferases. Enzyme Syst. that metabolise drugs other xenobiotics, 42. 219–252. 10.3389/fpls.2020.00364

[B74] Sies H. (2021). Hydrogen peroxide as a central redox signaling molecule in physiological oxidative stress: Oxidative eustress. Redox Biol 41, 613–619. 10.1016/j.redox.2016.12.035 PMC5256672 28110218

[B75] Sies H. Berndt C. Jones D. P. (2017). Oxidative stress. Annu. Rev. Biochem 86, 715–748. 10.1146/annurev-biochem-061516-045037 28441057

[B76] Smith M. D. Wertheim J. O. Weaver S. Murrell B. Scheffler K. Kosakovsky Pond S. L. (2015). Less is more: An adaptive branch-site random effects model for efficient detection of episodic diversifying selection. Mol. Biol. Evol 32, 1342–1353. 10.1093/molbev/msv022 25697341 PMC4408413

[B77] Srikrishna G. (2012). S100A8 and S100A9: New insights into their roles in malignancy. J. Innate Immun 4, 31–40. 10.1159/000330095 21912088 PMC3250655

[B78] Tian R. Seim I. Ren W. Xu S. Yang G. (2019). Contraction of the ROS scavenging enzyme glutathione S-transferase gene family in cetaceans. G3 9, 2303–2315. 10.1534/g3.119.400224 31092607 PMC6643896

[B79] Tian R. Wang Z. Niu X. Zhou K. Xu S. Yang G. (2016). Evolutionary genetics of hypoxia tolerance in cetaceans during diving. Genome Biol. Evol 8, 827–839. 10.1093/gbe/evw037 26912402 PMC4824146

[B80] Upadhyaya B. Tian X. Wu H. Lou M. F. (2015). Expression and distribution of thiol-regulating enzyme glutaredoxin 2 (GRX2) in porcine ocular tissues. Exp. Eye Res 130, 58–65. 10.1016/j.exer.2014.12.004 25479045 PMC4276450

[B81] Vázquez-Medina J. P. Soñanez-Organis J. G. Burns J. M. Zenteno-Savín T. Ortiz R. M. (2011). Antioxidant capacity develops with maturation in the deep-diving hooded seal. J. Exp. Biol 214, 2903–2910. 10.1242/jeb.057935 21832133 PMC3154117

[B82] Vázquez-Medina P. V. Zenteno-Savín T. Elsner R. (2006). Antioxidant enzymes in ringed seal tissues: Potential protection against dive-associated ischemia/reperfusion. Comp. Biochem. Physiol. C. Toxicol. Pharmacol 142, 198–204. 10.1016/j.cbpc.2005.09.004 16269268

[B83] Vázquez-Medina J. P. Zenteno-Savín T. Elsner R. Ortiz R. M. (2012). Coping with physiological oxidative stress: A review of antioxidant strategies in seals. J. Comp. Physiol. B 182, 741–750. 10.1007/s00360-012-0652-0 22327141 PMC3387341

[B84] Vázquez Medina P. V. Zenteno-Savín T. Elsner R. (2007). Glutathione protection against dive-associated ischemia/reperfusion in ringed seal tissues. J. Exp. Mar. Biol. Ecol 3452, 110–118. 10.1016/j.jembe.2007.02.003

[B85] Wätjen W. Fritsche E. (2010). Rolle des Fremdstoffmetabolismus in Pharmakologie und Toxikologie: Teil 2: Phase-II-Reaktionen. Apothekenmagazin 9, 6–14.

[B86] Yim H.-S. Cho Y. S. Guang X. Kang S. G. Jeong J.-Y. Cha S.-S. (2014). Minke whale genome and aquatic adaptation in cetaceans. Nat. Genet 46, 88–92. 10.1038/ng.2835 24270359 PMC4079537

[B87] Zenteno-Savın T. Clayton-Hernández E. Elsner R. (2002). Diving seals: Are they a model for coping with oxidative stress? Comp. Biochem. Physiol. C. Toxicol. Pharmacol 133, 527–536. 10.1016/s1532-0456(02)00075-3 12458181

[B88] Zenteno-Savín T. Elsner R. (2000). Differential oxidative stress in ringed seal tissues. Free. Radic. Biol. Med 29, S139.

[B89] Zenteno-Savín T. Elsner R. (1998). Seals and oxidative stress. Free Radic. Biol. Med 25, 42.9655520

