# Supplementary material for: Elevated antioxidant defence in the brain of deep-diving pinnipeds
Source: Front Physiol. 2022 Dec 16;13:1064476. doi: 10.3389/fphys.2022.1064476 (PMC9800987; doi:10.3389/fphys.2022.1064476)
Supplement: Supplementary file 1 [file DataSheet1.docx]

**Supplemental Tables**

**Suppl. Table 1a:** Primer sequences used in qPCR expression analyses for mice (Mmu), hooded seals (Ccr) and harp seals (Pgr).

| **Primer name** | **species** | **Primer sequence (5'-3')** |
| --- | --- | --- |
| SOD1_fw | Mmu, Ccr, Pgr | GACCTGGGCAATGTGACTGC |
| SOD1_rev | Mmu, Ccr, Pgr | CACCTTTGCCCAAGTCATCT |
| GPX3_fw | Mmu | CTCCTGAGACCAGCCAAGAC |
| GPX3_rev | Mmu | GAGCCTAAGCCTGAATGCAC |
| GPX3_fw | Ccr, Pgr | TTCTCGCACTCTCTACAGCA |
| GPX3_rev | Ccr, Pgr | AAACATGTGCGTGCGATTGT |
| GSTK1_fw | Mmu, Ccr, Pgr | CAAATATGGGGCYTTTGGGCT |
| GSTK1_rev | Mmu, Ccr, Pgr | AGGGCCCATCCACTTCTCT |
| GSTO1_fw | Mmu | TTGGAGCTCAAGGAGTGTCTA |
| GSTO1_rev | Mmu | TCAGAGCCCATAATCACAGG |
| GSTO1_fw | Ccr, Pgr | TGATTGTGTAGACCACACTCC |
| GSTO1_rev | Ccr, Pgr | AGCCCATAGTCACAGGCCT |
| GLRX2_fw | Mmu | GAAAGAACCGTTCCCAGGATA |
| GLRX2_rev | Mmu | TGATGAACCAGAGGCAGCAAT |
| GLRX2_fw | Ccr, Pgr | CTGGTGAAAAAACTGTACCAAG |
| GLRX2_rev | Ccr, Pgr | ACTGATGAACTAGTGGAAGCAA |
| TXNRD3_fw | Mmu | AATTCGACAACGAACGTGTGGT |
| TXNRD3_fw | Ccr, Pgr | TCGACAATTATCGGGTGATAGG |
| TXNRD3_rev | Mmu, Ccr, Pgr | CTAGCCTCAGCAGCCTTTCT |

**Suppl. Table 1b:** Primer efficiencies for every gene, presented by species and brain regions.

| **Gene symbol** | **Brain region** | **Species** | **Efficiency** |
| --- | --- | --- | --- |
| **GLRX2** | visual cortex | Ccr+Pgr | 0.98 |
| **GLRX2** | visual cortex | Mmu | 0.89 |
| **GLRX2** | cerebellum | Ccr+Pgr | 0.82 |
| **GLRX2** | cerebellum | Mmu | 0.90 |
| **GLRX2** | hippocampus | Ccr+Pgr | 0.92 |
| **GLRX2** | hippocampus | Mmu | 0.85 |
| **GPX3** | visual cortex | Ccr | 1.04 |
| **GPX3** | visual cortex | Pgr | 1.32 |
| **GPX3** | visual cortex | Mmu | 0.82 |
| **GPX3** | cerebellum | Ccr | 1.20 |
| **GPX3** | cerebellum | Pgr | 1.49 |
| **GPX3** | cerebellum | Mmu | 1.22 |
| **GPX3** | hippocampus | Ccr | 1.09 |
| **GPX3** | hippocampus | Pgr | 1.13 |
| **GPX3** | hippocampus | Mmu | 1.92 |
| **GSTK1** | visual cortex | Ccr+Pgr | 1.10 |
| **GSTK1** | visual cortex | Mmu | 1.13 |
| **GSTK1** | cerebellum | Ccr+Pgr | 1.01 |
| **GSTK1** | cerebellum | Mmu | 1.20 |
| **GSTK1** | hippocampus | Ccr+Pgr | 1.25 |
| **GSTK1** | hippocampus | Mmu | 1.29 |
| **GSTO1** | visual cortex | Ccr+Pgr | 0.94 |
| **GSTO1** | visual cortex | Mmu | 1.27 |
| **GSTO1** | cerebellum | Ccr+Pgr | 0.84 |
| **GSTO1** | cerebellum | Mmu | 0.85 |
| **GSTO1** | hippocampus | Ccr+Pgr | 1.10 |
| **GSTO1** | hippocampus | Mmu | 0.90 |
| **RBFOX3** | visual cortex | Ccr | 0.97 |
| **RBFOX3** | visual cortex | Pgr | 0.95 |
| **RBFOX3** | visual cortex | Mmu | 1.11 |
| **RBFOX3** | cerebellum | Ccr | 1.01 |
| **RBFOX3** | cerebellum | Pgr | 1.06 |
| **RBFOX3** | cerebellum | Mmu | 1.19 |
| **RBFOX3** | hippocampus | Ccr | 1.07 |
| **RBFOX3** | hippocampus | Pgr | 0.96 |
| **RBFOX3** | hippocampus | Mmu | 1.03 |
| **SOD1** | visual cortex | Ccr+Pgr | 1.30 |
| **SOD1** | visual cortex | Mmu | 1.61 |
| **SOD1** | cerebellum | Ccr+Pgr | 1.23 |
| **SOD1** | cerebellum | Mmu | 1.47 |
| **SOD1** | hippocampus | Ccr+Pgr | 1.15 |
| **SOD1** | hippocampus | Mmu | 1.38 |
| **TXNRD3** | visual cortex | Ccr+Pgr | 1.47 |
| **TXNRD3** | visual cortex | Mmu | 2.23 |
| **TXNRD3** | cerebellum | Ccr+Pgr | 1.23 |
| **TXNRD3** | cerebellum | Mmu | 1.16 |
| **TXNRD3** | hippocampus | Ccr+Pgr | 1.36 |
| **TXNRD3** | hippocampus | Mmu | 1.13 |

**Suppl. Table 2:** Expression of 49 antioxidant genes in neurons of the visual cortex of the mouse (*Mus musculus*, n = 3) and the hooded seal (*Cystophora cristata*, n = 3). Gene expression is presented as TPM (transcripts per million) values and the RNA-seq data were extracted from Geßner et al. (2022). Expression values of genes in the GO term „antioxidant activity“ (GO:0016209) were taken from Geßner et al. (2022), while expression values of genes of the GO term „glutathione metabolic process“ (GO:0006749) and from a list of human antioxidants (Gelain et al. 2009) were extracted in this study. Since some genes occur in more than in one GO term, we have listed them only once to avoid repetition.

| **Annotated Term** | **Gene symbol** | **Fold change** | **FDR** | **TPM mouse** | **TPM hooded seal** |
| --- | --- | --- | --- | --- | --- |
| **antioxidant activity (GO:0016209)** taken from Geßner et al (2022) | | | | | |
| glutathione peroxidase activity | *ALOX5AP* | 21.5 | 2.21E-29 | 0.5 | 12.7 |
|  | *GPX1* | 2.2 | 2.52E-05 | 65.4 | 140.2 |
|  | *GPX3* | 8.6 | 1.47E-11 | 4.0 | 34.2 |
|  | *GSTK1* | 26.9 | 6.76E-72 | 0.7 | 19.1 |
|  | *GSTO1* | 15.1 | 2.30E-30 | 3.6 | 53.9 |
|  | *GSTO2* | 9.9 | 2.12E-07 | 0.1 | 1.1 |
|  | *LTC4S* | -8.9 | 1.77E-07 | 1.8 | 0.2 |
|  | *MGST1** | 13.6 | 4.86E-21 | 0.2 | 1.3 |
|  | *PTGES* | 4.5 | 1.50E-03 | 0.3 | 1.1 |
|  | *PTGS2* | -2.4 | 7.40E-03 | 2.9 | 1.2 |
| glutathione-disulfide reductase (NADPH) activity | *GSR* | 2.0 | 1.50E-02 | 6.5 | 12.8 |
| bromide peroxidase | *PXDN* | -2.5 | 3.69E-07 | 2.3 | 0.9 |
| antioxidant activity | *PRDX2* | 3.4 | 3.24E-16 | 114.1 | 388.0 |
|  | *PRXL2A* | 3.5 | 6.17E-15 | 7.9 | 27.8 |
|  | *PRXL2B* | -4.3 | 1.35E-19 | 35.3 | 8.2 |
|  | *S100A9* | 99.7 | 5.00E-03 | 0.1 | 6.1 |
|  | *SELENOW* | 2.0 | 4.00E-02 | 15.0 | 30.1 |
|  | *SOD1* | 9.1 | 7.29E-59 | 52.8 | 481.0 |
| superoxide dismutase activity | *NQO1* | 10.1 | 6.35E-24 | 1.5 | 15.2 |
| catalase activity | *CYGB* | -2.7 | 8.72E-08 | 12.3 | 4.6 |
| thioredoxin-disulfide reductase activity | *SELENOT* | 17.6 | 6.79E-75 | 2.3 | 41.5 |
|  | *TXNRD1* | 2.6 | 1.34E-06 | 7.6 | 20.0 |
|  | *TXNRD3* | 4.7 | 6.03E-07 | 1.3 | 5.9 |
| **glutathione metabolic process (GO:0006749)** | | | | | |
| glutathione metabolic process | *CTH* | 10.6 | 9.03E-11 | 0.8 | 8.4 |
|  | *CTNS* | -2.2 | 6.40E-04 | 2.2 | 1.0 |
|  | *ETHE1* | 2.1 | 1.10E-02 | 16.0 | 33.7 |
|  | *GLO1* | 8.3 | 5.26E-28 | 8.4 | 70.3 |
|  | *GSTM1* | -69.7 | 4.85E-53 | 9.5 | 0.1 |
|  | *GSTM3* | -35.4 | 1.42E-111 | 50.1 | 1.4 |
|  | *GSTM4* | -9.4 | 1.75E-06 | 1.5 | 0.1 |
|  | *GSTZ1* | -2.9 | 1.07E-05 | 1.9 | 0.7 |
|  | *NAT8* | -17.3 | 2.38E-09 | 2.4 | 0.1 |
|  | *NFE2L1* | -4.1 | 0 | 64.9 | 15.7 |
| glutathione biosynthetic process | *GCLM* | -5.7 | 3.38E-26 | 10.6 | 1.8 |
|  | *GSS* | 2.3 | 1.30E-03 | 3.9 | 9.1 |
|  | *SLC1A2* | 3.5 | 9.08E-11 | 12.5 | 43.8 |
| positive regulation of biosynthesis | *NFE2L2* | 15.2 | 4.32E-13 | 0.7 | 10.3 |
| glutathione biosynthetic & catabolic process | *GGT7* | -2.8 | 7.46E-14 | 133.5 | 47.5 |
| glutathione catabolic process | *CHAC2* | -2.9 | 8.62E-06 | 5.0 | 1.7 |
| **Human antioxidant genes (Gelain et al 2009)** | | | | | |
| Thiol redox | *GLRX* | -3.7 | 2.79E-07 | 1.2 | 0.3 |
|  | *GLRX2* | -3.3 | 1.09E-08 | 3.8 | 1.1 |
|  | *GLRX3* | -2.4 | 3.12E-12 | 39.8 | 16.7 |
|  | *MT1A* | 10.0 | 1.10E-02 | 0.1 | 1.7 |
|  | *MT1M* | -105.9 | 6.25E-54 | 66.2 | 0.6 |
|  | *MT2A* | -49.3 | 4.76E-46 | 20.2 | 0.4 |
|  | *TXNDC5* | -2.0 | 4.60E-04 | 2.5 | 1.3 |
|  | *TXNIP* | 8.5 | 8.46E-14 | 2.3 | 19.3 |
| **additional genes with known antioxidant activity** | | | | | |
| antioxidant defence | *HMOX2* | 2.5 | 3.94E-07 | 40.3 | 99.0 |
| antioxidant defence | *PON2* | 4.0 | 3.55E-14 | 12.0 | 48.6 |

**Suppl. Table 3:** GenBank accession numbers of genes tested for positive selection. Sequences of the hooded seal were extracted from Geßner et al (2022) who deposited sequence files at the NCBI Sequence Read Archive (PRJNA785765). Dash (-) indicates missing sequences.

|  |  |  |  |  |  |  |  |
| --- | --- | --- | --- | --- | --- | --- | --- |
| **Species** | **SOD1** | **GPX3** | **GSTO1** | **GSTK1** | **GLRX2** | **TXNRD3** | **GSR** |
| Gray seal | XM_036070661.1 | XM_036090652.1 | XM_036072388.1 | XM_036095820.1 | - | XM_036078530.1 | XM_036107086.1 |
| Hawaiian monk seal | XM_021678086.1 | XM_021700997.1 | XM_021700263.1 | XM_021692779.1 | XM_021686656.1 | XM_021695011.1 | XM_044911945.1 |
| Southern elephant seal | XM_035017588.1 | XM_034999581.1 | XM_035028644.1 | XM_035021296.1 | - | XM_045904193.1 | XM_045879790.1 |
| Harbour seal | XM_032393272.1 | XM_032395275.1 | XM_032423193.1 | XM_032401423.1 | XM_032422059.1 | XM_032412074.1 | XM_032413462.1 |
| Weddell seal | XM_031041374.1 | XM_006752411.2 | XM_031040229.1 | XM_006749538.1 | XM_031041961.1 | XM_031018881.1 | XM_031026276.1 |
| Walrus | XM_004406294.1 | XM_004402830.2 | - | XM_004414576.1 | - | - | XM_004408281.2 |
| Dog | NM_001003035.1 | NM_001164454.1 | XM_038440499.1 | XM_005629528.4 | XM_038448378.1 | NM_001122778.1 | XM_038689973.1 |
| Ferret | XM_013058840.1 | XM_004737800.2 | XM_013054574.1 | XM_004782546.2 | XM_045081144.1 | XM_004738666.3 | XM_045073784.1 |
| Polar Bear | XM_040621040.1 | XM_040630394.1 | XM_040623945.1 | XM_008697303.2 | XM_040636025.1 | XM_040628356.1 | XM_040643282.1 |
| Grizzly Bear | XM_026514338.1 | XM_026510805.1 | XM_026494065.1 | XM_026512905.1 | XM_044390677.1 | XM_026501505.2 | XM_026508378.2 |
| Giant Panda | XM_002928799.4 | XM_002920757.4 | XM_034663250.1 | XM_002924165.4 | XM_011230064.3 | XM_034657258.1 | XM_034647544.1 |

**Suppl. Table 4:** Antioxidant genes in pinnipeds show no signatures of positive selection when compared to non-diving carnivores using two models (BUSTED, aBSREL). Only when both models inferred positive selection were genes considered to have been positively selected.

|  | **BUSTED** | | **aBSREL** | |
| --- | --- | --- | --- | --- |
| **gene symbol** | **p-value** | **alignment (%)** | **p-value** | **branch** |
| GPX3 | 0.5 | - | - | - |
| GSTO1 | 0.5 | - | < 0.001 | gray seal |
| GSTK1 | 0.5 | - | - | - |
| SOD1 | 0.056 | - | - | - |
| GSR | <0.001 | 3,75 | - | - |
| TXNRD3 | 0.058 | - | 0.002 | hooded seal |
| GLRX2 | 0.304 | - | - | - |

**Suppl. Table 5:** All glutathione-s-transferases (GSTs) present in the hooded seal neuronal transcriptome with a TPM-value ≥ 1 in either species, mouse or hooded seal.

| **Gene symbol** | **Fold change** | **FDR** | **TPM mouse** | **TPM hooded seal** |
| --- | --- | --- | --- | --- |
| GSTK1 | 26.9 | 6.76E-72 | 0.7 | 19.1 |
| GSTM1 | -69.7 | 4.85E-53 | 9.5 | 0.1 |
| GSTM3 | -35.4 | 1.42E-111 | 50.1 | 1.4 |
| GSTM4 | -9.4 | 1.75E-06 | 1.5 | 0.1 |
| GSTM5 | -745.3 | 2.07E-71 | 6.0 | 0.0 |
| GSTO1 | 15.1 | 2.30E-30 | 3.6 | 53.9 |
| GSTO2 | 9.9 | 2.12E-07 | 0.1 | 1.1 |
| GSTZ1 | -2.9 | 1.07E-05 | 1.9 | 0.7 |
| **SUM** |  |  | **73.5** | **76.5** |

**Supplemental Figures**

**Suppl. Figure 1:** Glutathione reductase (GSR) amino-acid alignment constructed using MAFFT (Katoh and Standley, 2013) and visualized with AliView (Larsson, 2014). The alignment positions of three pinniped-specific amino-acid substitutions are indicated by red arrows.


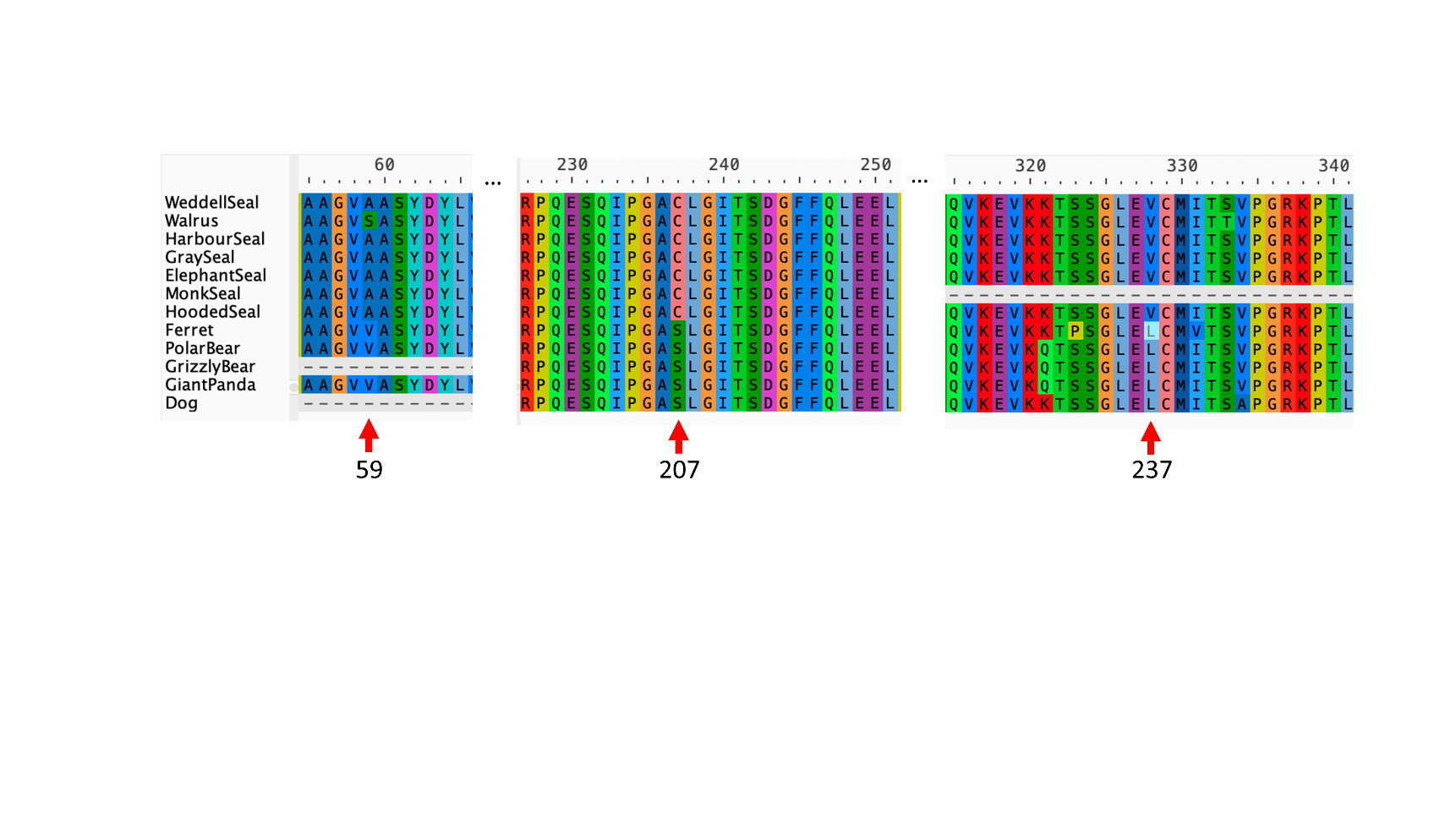


Reference list:

Katoh, K., and D.M. Standley. 2013. MAFFT multiple sequence alignment software version 7: improvements in performance and usability. *Molecular biology and evolution*. 30:772-780.

Larsson, A. 2014. AliView: a fast and lightweight alignment viewer and editor for large datasets. *Bioinformatics*. 30:3276-3278.
